# Supplementary material for: Myomerger-derived peptide enhances skeletal muscle tropism and reduces liver transduction of lipid nanoparticles for gene delivery
Source: Mol Ther Nucleic Acids. 2025 Nov 27;37(1):102785. doi: 10.1016/j.omtn.2025.102785 (PMC12741376; doi:10.1016/j.omtn.2025.102785)
Supplement: Document S1. Figures S1–S7 and Tables S1–S3 [file mmc1.pdf]

OMTN, Volume *37*

Supplemental information

Myomerger-derived peptide enhances skeletal muscle tropism and reduces liver transduction of lipid nanoparticles for gene delivery

Jacqueline Ji, Eva Lipkow, Nicolas Anton, Corinne Crucifix, Pascal Eberling, and Jocelyn Laporte

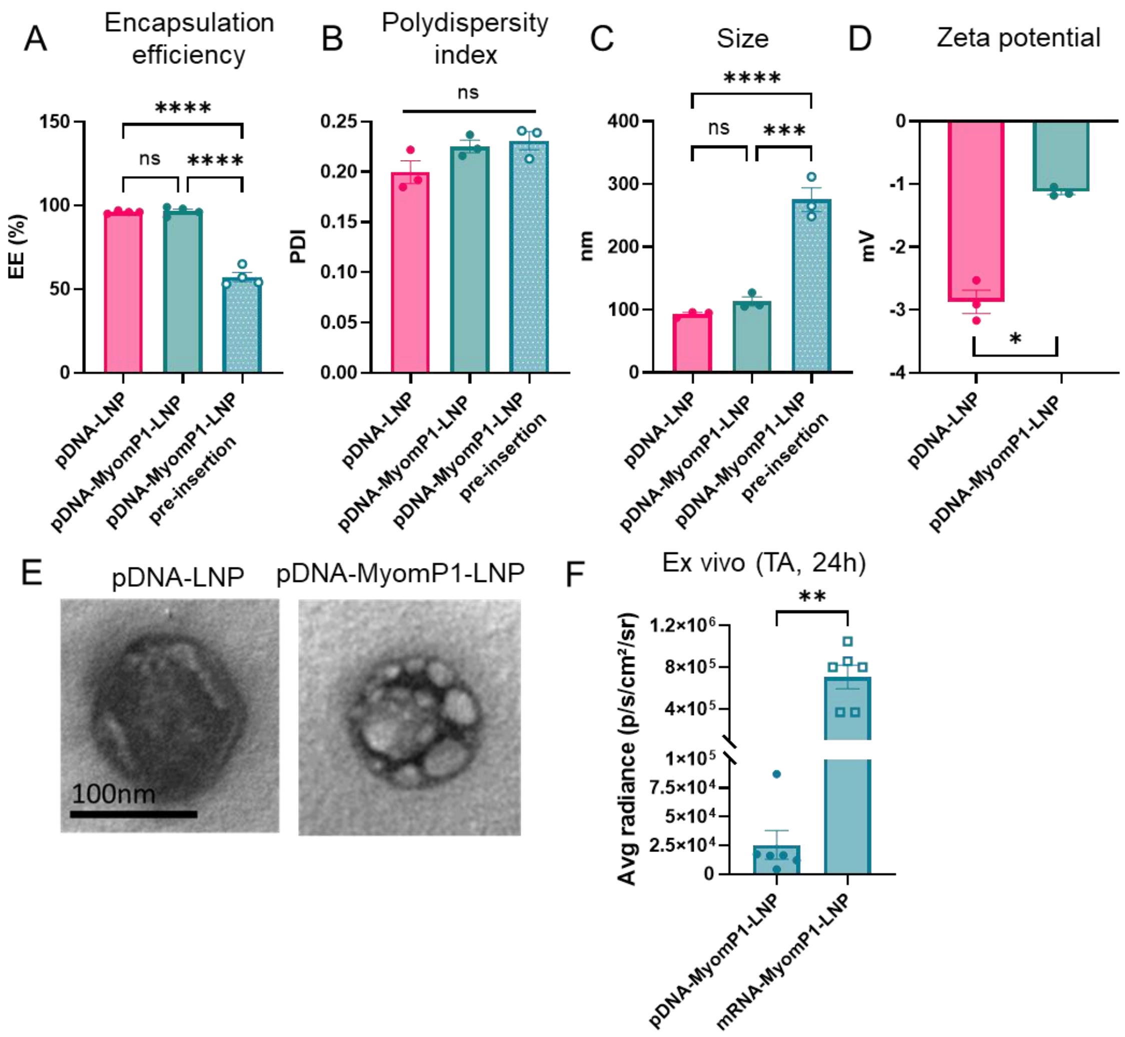

Figure S1. Characterization of peptide-functionalized pDNA-LNPs. pDNA-MyomP1-LNP refers to the LNP formulation in which DSPE-PEG(2000)-DBCO/MyomP1 is incorporated after LNP formation (post-insertion). In contrast, pDNA-MyomP1-LNP with pre-insertion indicates that MyomP1 is incorporated by incubating it with LNPs that already contain DBCO during the formulation process. (**A**) Encapsulation efficiency of pDNA-LNPs. Data are presented as mean ± SEM; ****$p<0.0001$. (**B)** Polydispersity index of pDNA-LNPs. Data is presented as the mean ± SEM. (**C)** Size of pDNA-LNPs. Data are presented as mean ± SEM; ***$p<0.001$, ****$p<0.0001$. (**D)** Zeta potential of pDNA-LNPs. The zeta potential was measured at pH=7 in PBS buffer. Data are presented as mean ± SEM; *$p<0.05$. (**E)**

Transmission electron microscopy images of pDNA-LNPs. Scale bar is indicated. (**F**) Ex vivo quantification of bioluminescence from TA muscles 24h after pDNA-MyomP1-LNP or mRNA-MyomP1-LNP injection. n= 6; Data are presented as mean ± SEM; **p<0.01.

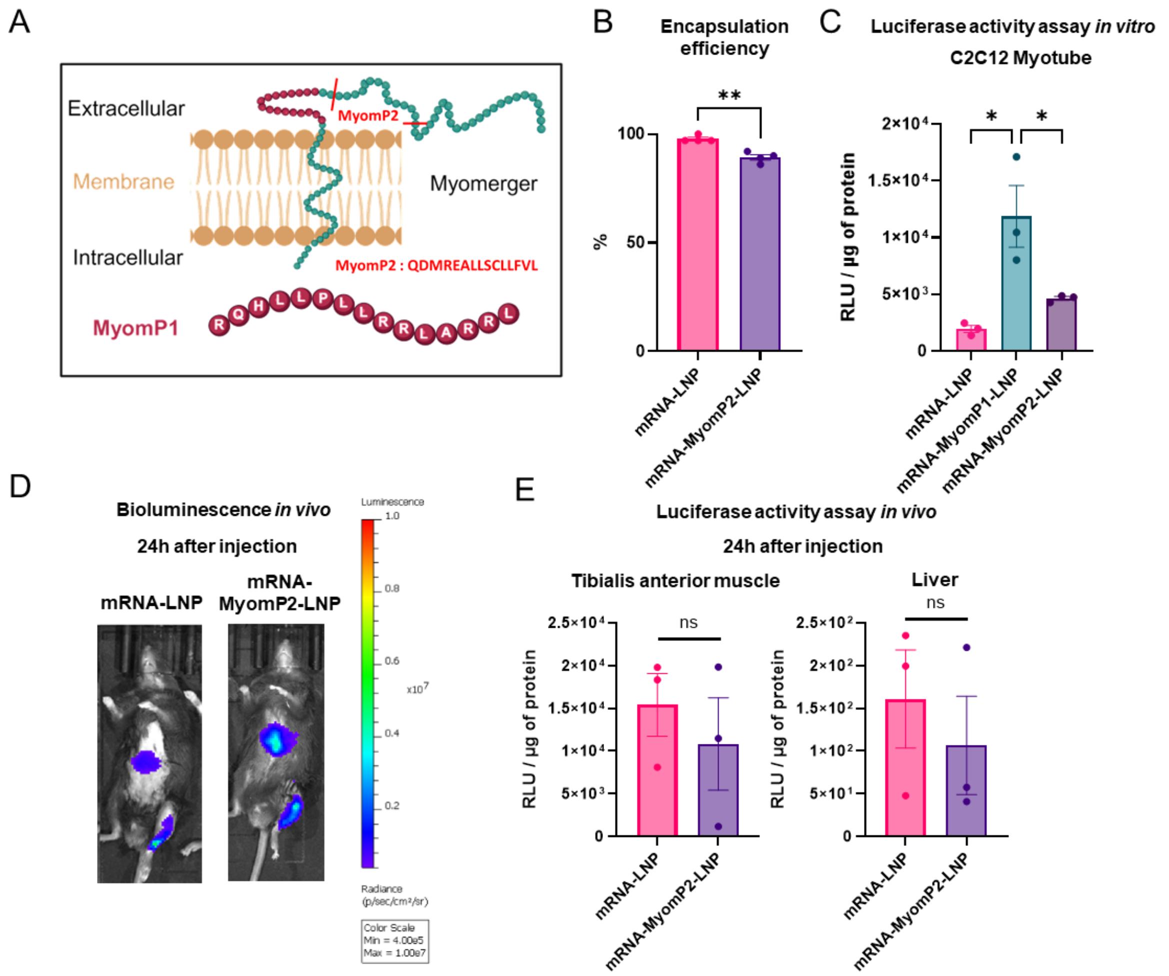

Figure S2. **MyomP2 functionalization shows no enhanced transduction efficiency or off-target effects *in vitro* and *in vivo*.** (**A**) Illustration of the peptide MyomP2 compared to MyomP1. Sequence are indicated in blue and red respectively. (**B**) Encapsulation efficiency. (**C**) Luciferase activity in C2C12 myotubes after transduction with mRNA-LNPs. Data are presented as mean ± SEM; *p<0.05. (**D**) *In vivo* bioluminescence imaging at 24 h post-injection of mRNA-LNPs in C57BL/6 mice. mRNA-LNPs was injected intramuscularly in the TA at the dose of 1 µg. Color scale indicates radiance (photons/s/cm²/sr). (**E**) Luciferase activity in TA

and liver at 24 h shown as RLU/μg of protein. n=3; One-way ANOVA followed by Fisher's LSD test; Data are presented as mean ± SEM; *p<0.05 **p<0.01.

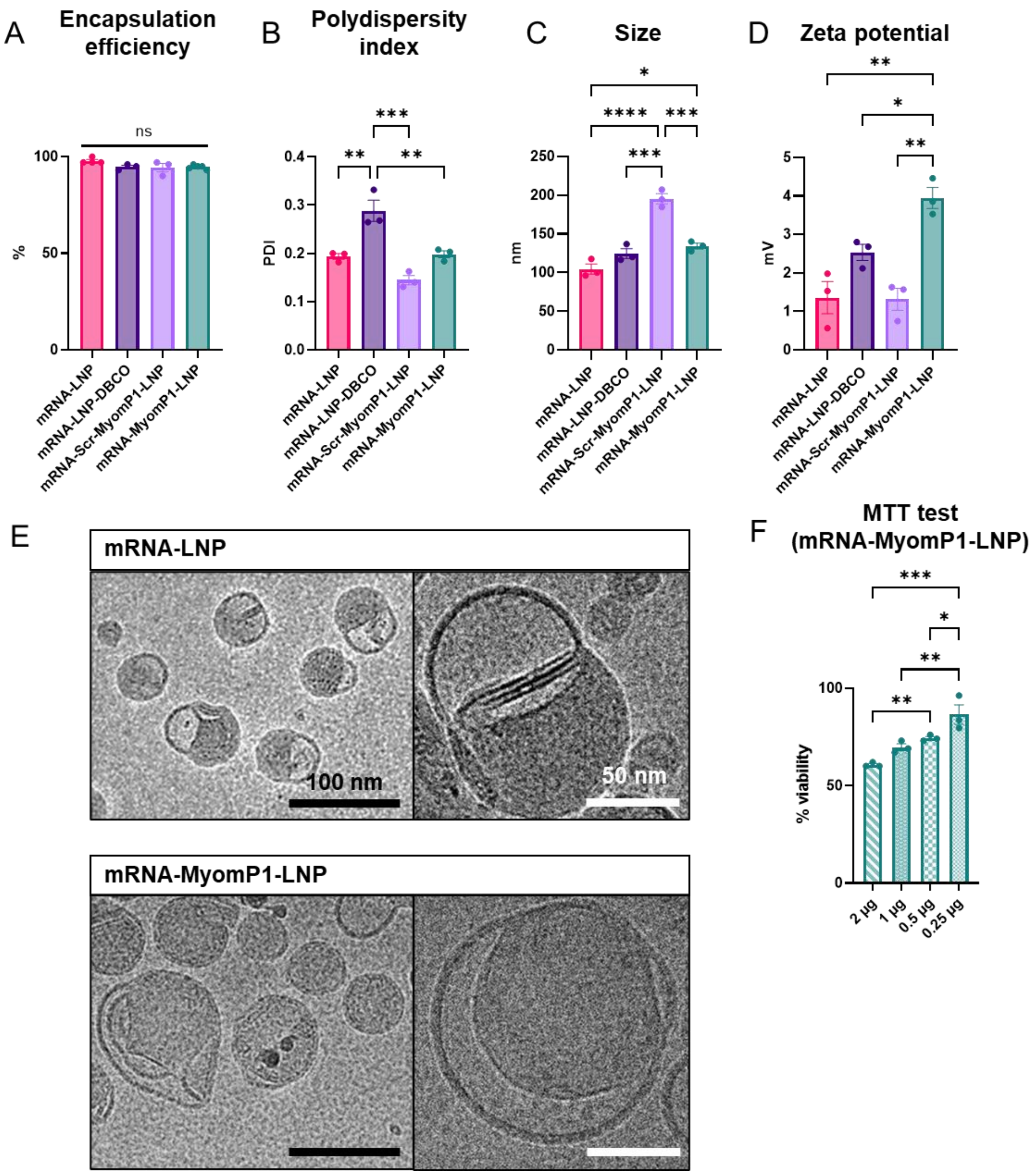

Figure S3. Characterization of peptide-functionalized mRNA-LNPs. (A) Encapsulation efficiency of mRNA-LNPs. **(B)** Polydispersity index of mRNA-LNPs. **(C)** Size of mRNA-LNPs. **(D)** Zeta potential of mRNA-LNPs. The zeta potential was measured at pH=7 in PBS buffer. **(E)** Morphology of mRNA-LNPs. Cryo-electron microscopy images were generated to

visualize mRNA-LNP morphology. Black scale bars represent 100 nm and 50 nm. (**F**) Cell viability in myoblasts after transduction with different doses of mRNA-MyomP1-LNP. n=3; One-way ANOVA followed by Fisher's LSD test; Data are presented as mean ± SEM; *p<0.05 **p<0.01, ***p<0.001, ****p<0.0001.

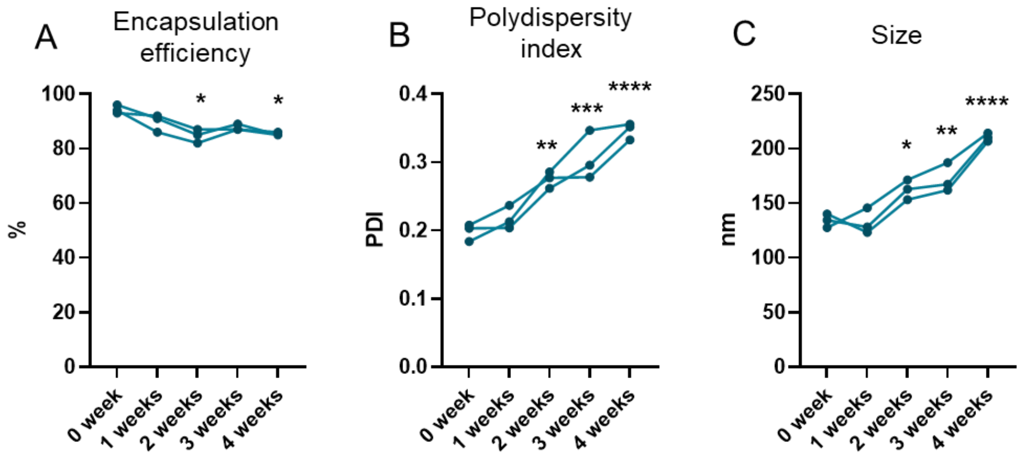

Figure S4. Evaluation of the stability of mRNA-MyomP1-LNP over weeks at 4°C. The encapsulation efficiency, polydispersity index, and particle size of mRNA-MyomP1-LNPs were evaluated weekly for up to four weeks during storage at 4°C. **(A)** Encapsulation efficiency of mRNA-MyomP1-LNPs. **(B)** Polydispersity index of mRNA-MyomP1-LNPs. **(C)** Size of mRNA-MyomP1-LNPs. n=3; One-way ANOVA followed by Fisher's LSD test; Data are presented as mean ± SEM; *p<0.05 **p<0.01, ***p<0.001, ****p<0.0001; *vs 0 week.

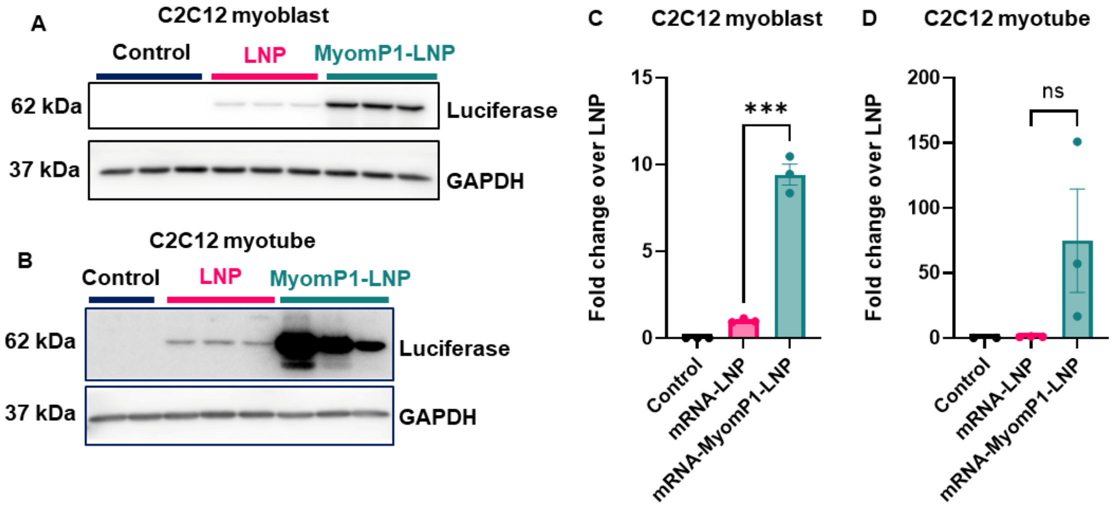

Figure S5. Luciferase protein level in C2C12 myoblasts and myotubes transfected with mRNA-LNPs. (A,B) Luciferase protein expression in transfected-C2C12 myoblasts (**A**) and myotubes (**B**). **(C,D)** Protein level was normalized with GAPDH expression. n=3; One-way ANOVA followed by Fisher's LSD test or Krustal-Wallis test followed by Dunn's LSD test; Data are presented as mean ± SEM; ***p<0.001.

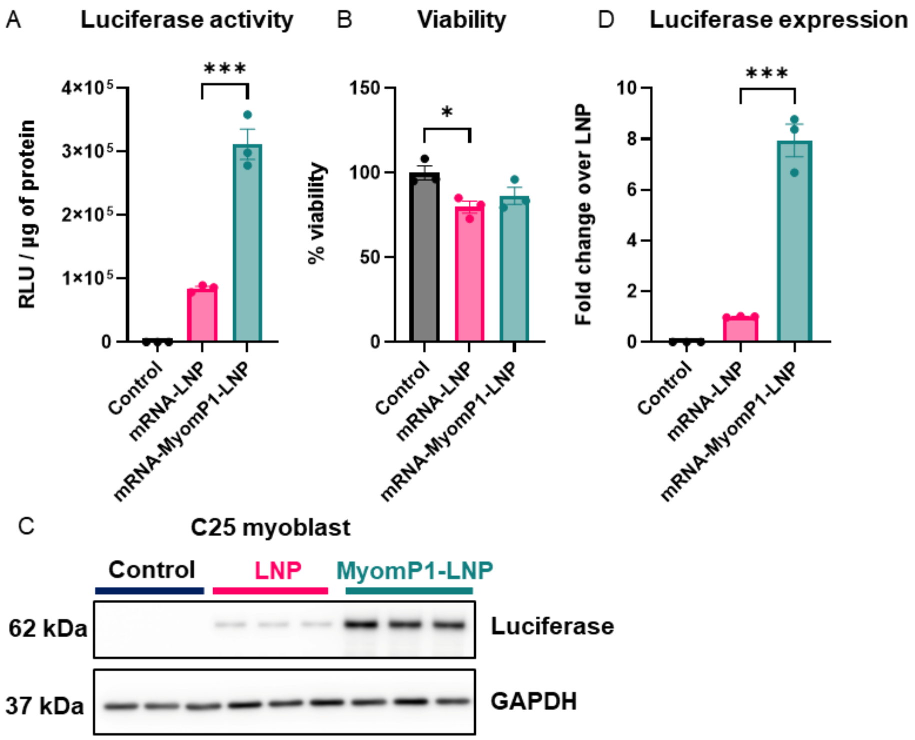

Figure S6. ***In vitro*** **transfection efficacy and cell viability of peptide-functionalized mRNA-LNPs in human myoblasts. (A)** Luciferase activity in C25 human myoblasts shown as relative luminescence units (RLU) per µg of total protein. **(B)** Viability of C25 human myoblasts post-transduction. **(C,D)** Luciferase protein expression in transfected C25 human myoblasts. Protein level was normalized with GAPDH expression. n=3; Data are presented as mean ± SEM; One-way ANOVA followed by Fisher's LSD test; *p < 0.05, ***p < 0.001.

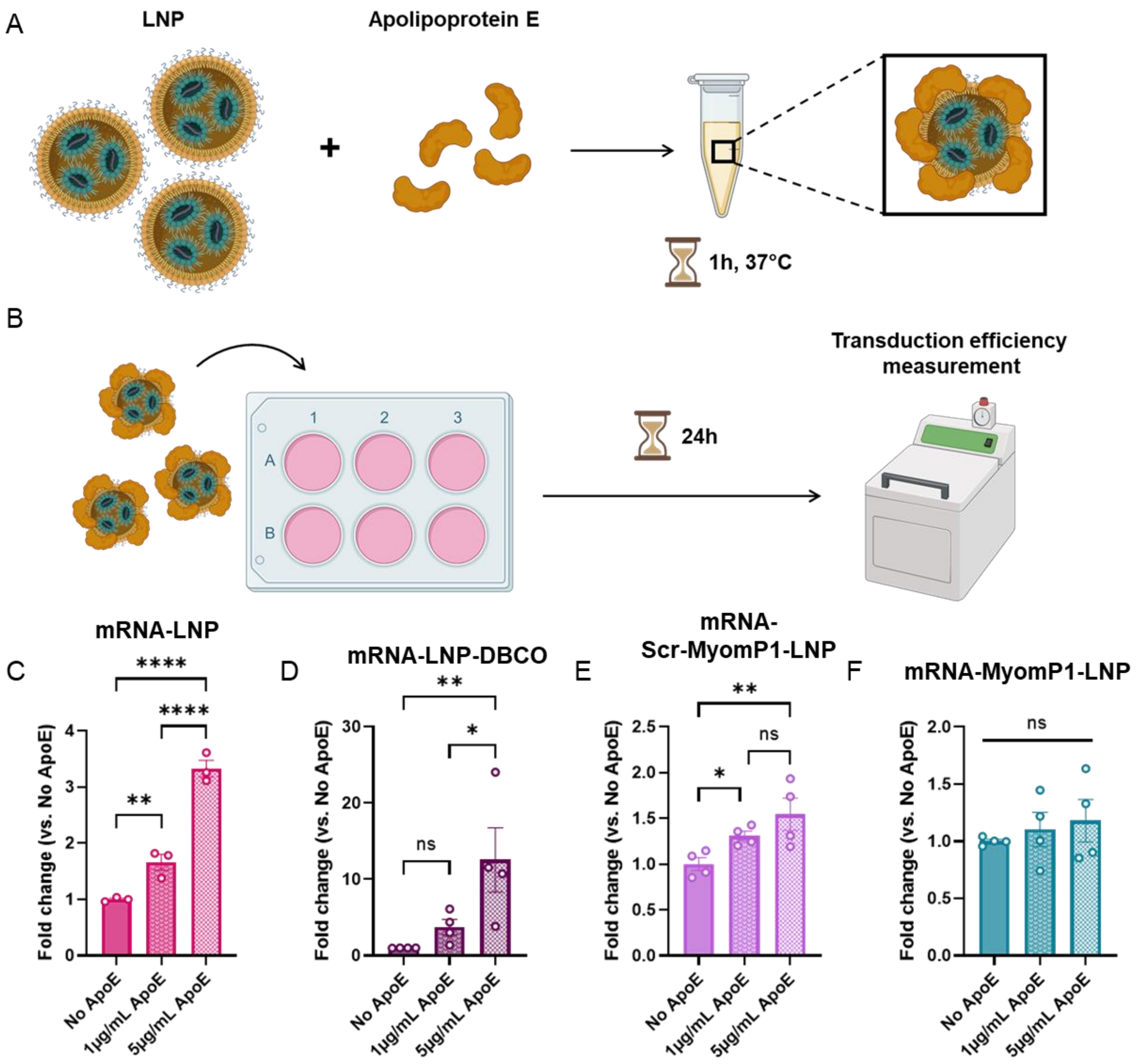

Figure S7. Impact of ApoE on mRNA-LNP transduction efficiency. (**A**) Schematic representation of ApoE corona formation on the surface of LNPs. (**B**) Illustration of the *in vitro*

protocol in C2C12 myoblasts to assess the effect of ApoE on transduction efficiency. (C-F) Luciferase activity in C2C12 myoblasts after incubation with mRNA-LNPs in the absence or presence of ApoE at different doses (1 or 5 µg/mL) for: (C) mRNA-LNP, (D) mRNA-LNP-DBCO, (E) mRNA-Scr-MyomP1-LNP, and (F) mRNA-MyomP1-LNP. Luciferase activity is expressed as fold change relative to the No ApoE condition. Data represent mean ± SEM, n = 3-4; One-way ANOVA followed by Fisher's LSD test; *$p < 0.05$, **$p < 0.01$, ****$p < 0.0001$.

Table S1. Lipid composition of LNPs

Lipid	Stock solution (mg/mL)	Molar ratio	CAS number	Reference
Dlin-KC2-DMA	100	50	1190197-97-7	MedChemExpress, #HY-112758
SM-102	100		2089251-47-6	MedChemExpress, #HY-134541
1,2-DSPC	25	10	816-94-4	MedChemExpress, #HY-W040193
Cholesterol	5	38.5	57-88-5	MedChemExpress, #HY-N0322
DMG-PEG2000	1	1.4	160743-62-4	MedChemExpress, #HY-112764
DSPE-PEG2000-DBCO	1	0.1	2052955-83-4	Avanti, #880229

Table S2. Primers used for genes encoding immune-related markers and the housekeeping gene

Gene	Forward	Reverse
Il-6	ACAACCACGGCCTTCCCTACTT	CACGATTTCCCAGAGAACATGTG
Irf7	CCCATCTTCGACTTCAGCAC	TGTAGTGTGGTGACCCTTGC
Ccl2	AGGTCCCTGTCATGCTTCTG	AAGGCATCACAGTCCGAGTC
Cxcl10	CCAAGTGCTGCCGTCATTTTC	TCCCTATGGCCCTCATTCTCA
Rps11	CGCGTGGTGAATAAGGAAGC	GTAAGCACGCTCCGTCTGAA

Table S3. Antibodies used in flow cytometry

Name	Format	Clone	Supplier	Reference	Dilution
CD4	PE	RM4-5	BD Biosciences	553049	1/100e
CD8	Alexa 700	53-6.7	BD Biosciences	557959	1/100e
CD44 (Pgp-1)	FITC	IM7.8.1	BD Biosciences	561859	1/100e
CD62L	PE-CF594	MEL-14	BD Biosciences	562404	1/100e
CD19	RB705	1D3	BD Biosciences	570651	1/200e
B220	Pe-Cy7	RAR3-6B2	e-Bioscience	25-0452	1/100e
