# Supplementary material for: Myomerger-derived peptide enhances skeletal muscle tropism and reduces liver transduction of lipid nanoparticles for gene delivery
Source: Mol Ther Nucleic Acids. 2025 Nov 27;37(1):102785. doi: 10.1016/j.omtn.2025.102785 (PMC12741376; doi:10.1016/j.omtn.2025.102785)
Supplement: Document S2. Article plus supplemental information [file mmc2.pdf]

Molecular Therapy
Nucleic Acids
Original Article

Myomerger-derived peptide enhances skeletal muscle tropism and reduces liver transduction of lipid nanoparticles for gene delivery

Jacqueline Ji,[1] Eva Lipkow,[1] Nicolas Anton,[2] Corinne Crucifix,[3] Pascal Eberling,[4] and Jocelyn Laporte[1]

[1]Département de Médecine Translationelle, Institut de Génétique et de Biologie Moléculaire et Cellulaire (IGBMC), Cnrs UMR7104, Inserm U1258, Université de Strasbourg, 67404 Illkirch-Graffenstaden, France; [2]INSERM (French National Institute of Health and Medical Research), UMR 1260, Regenerative Nanomedicine (RNM), FMTS, Université de Strasbourg, 67000 Strasbourg, France; [3]Centre for Integrative Biology (CBI), Department of Integrated Structural Biology, IGBMC, CNRS, Inserm, Université de Strasbourg, 67404 Illkirch-Graffenstaden, France; [4]Plateforme de Protéomique, Institut de Génétique et de Biologie Moléculaire et Cellulaire (IGBMC), Cnrs UMR7104, Inserm U1258, Université de Strasbourg, 67404 Illkirch-Graffenstaden, France

Lipid nanoparticles (LNPs) are emerging as nonviral vectors for gene therapy; yet, their strong liver tropism and lack of tissue specificity remain limiting. Here, we developed, through rational design, a skeletal muscle-targeted delivery platform by functionalizing LNPs with MyomP1, an extracellular conserved peptide derived from the muscle-specific fusogenic protein Myomerger. MyomP1-LNPs were engineered to encapsulate plasmid DNA or mRNA. *In vitro*, MyomP1 conjugation significantly increased transduction efficiency in murine and human myoblasts and myotubes. *In vivo*, MyomP1-LNPs significantly enhanced muscle transduction when delivering DNA cargo, strongly reduced liver accumulation following intramuscular and intravenous mRNA delivery, and attenuated local immune activation. This work demonstrates a ligand-guided strategy to overcome organ-specific barriers in nonviral gene transfer, with improved safety and specificity. It suggests that MyomP1-engineered LNPs hold strong potential to improve therapeutic outcomes for patients with rare muscle diseases, offering a promising alternative to traditional viral gene therapy platforms.

INTRODUCTION

Gene therapy and gene modulation are the main therapeutic strategies to tackle inherited and acquired diseases.[1–4] However, in the case of muscle disorders, delivering genetic material to the entire skeletal muscle system remains a considerable challenge, as this tissue represents approximately 40% of the total body mass in mammals. Currently, adeno-associated virus (AAV) vectors are the most commonly used carriers for gene delivery in inherited muscle disorders.[4–7] Notably, ELEVIDYS, an AAV-based gene therapy for Duchenne muscular dystrophy (DMD), represents the first FDA (Food, Drug and Administration)-approved viral vector-based gene therapy for this disease.[8] While AAVs are very attractive in muscle gene delivery, several critical limitations persist. These include limited cargo size, pre-existing immunity against AAV capsids, and the complexity and high cost of vector production.[5,9–13] Additionally, off-target distribution in nonmuscle organs, such as the liver, is a concern. Indeed, this limitation was tragically demonstrated in the clinical trial NCT03199469, in which AAV8-*MTM1* gene therapy for severe myotubular myopathy led to fatal hepatic complications in four patients.[10,11,14]

In response to these challenges, nonviral vectors have emerged as an alternative to viral vectors. Among them, lipid nanoparticles (LNPs) have gained popularity due to their modularity, scalable production, and clinical success.[15,16] These vectors are composed of a mixture of lipids, including ionizable lipids for nucleic acid encapsulation, phospholipids and cholesterol for stability, and polyethylene glycol (PEG) lipids for size and clearance kinetics control.[17] ONPATTRO, the first FDA-approved small interfering RNA-LNP therapy, and two severe acute respiratory syndrome coronavirus 2 (SARS-CoV-2) mRNA vaccines underscore the therapeutic potential of this platform. However, translating LNPs to gene therapies targeting skeletal muscle remains challenging, and several parameters still need to be investigated.[18,19] After local or systemic administration, LNPs quickly bind apolipoprotein E (ApoE), thereby facilitating uptake by the liver via the low-density lipoprotein receptor (LDLR).[20] Additionally, LNPs are known to trigger potent innate and adaptive immune responses, which vary depending on the nucleic acid cargo.[21–23] To overcome these limitations, strategies such as passive and active targeting have been investigated. For example, modifying the ionizable lipid can reduce liver and spleen uptake following intramuscular injection.[24] Selective organ targeting (SORT), achieved by altering lipid composition, further enhances tissue specificity.[25] Moreover, ligand-mediated targeting has demonstrated high potency in redirecting organ transduction. Conjugating muscle-homing peptides to polymeric nanoparticles, or decorating LNPs with muscle-receptor-binding antibodies, has shown promise for increased muscle delivery.[26,27] Finally, recent

Received 15 July 2025; accepted 24 November 2025;
https://doi.org/10.1016/j.omtn.2025.102785.

Correspondence: Jocelyn Laporte, Département de Médecine Translationelle, Institut de Génétique et de Biologie Moléculaire et Cellulaire (IGBMC), Cnrs UMR7104, Inserm U1258, Université de Strasbourg, 67404 Illkirch-Graffenstaden, France.
E-mail: jocelyn@igbmc.fr

Published by Elsevier Inc. on behalf of The American Society of Gene and Cell Therapy.
This is an open access article under the CC BY-NC-ND license (http://creativecommons.org/licenses/by-nc-nd/4.0/).

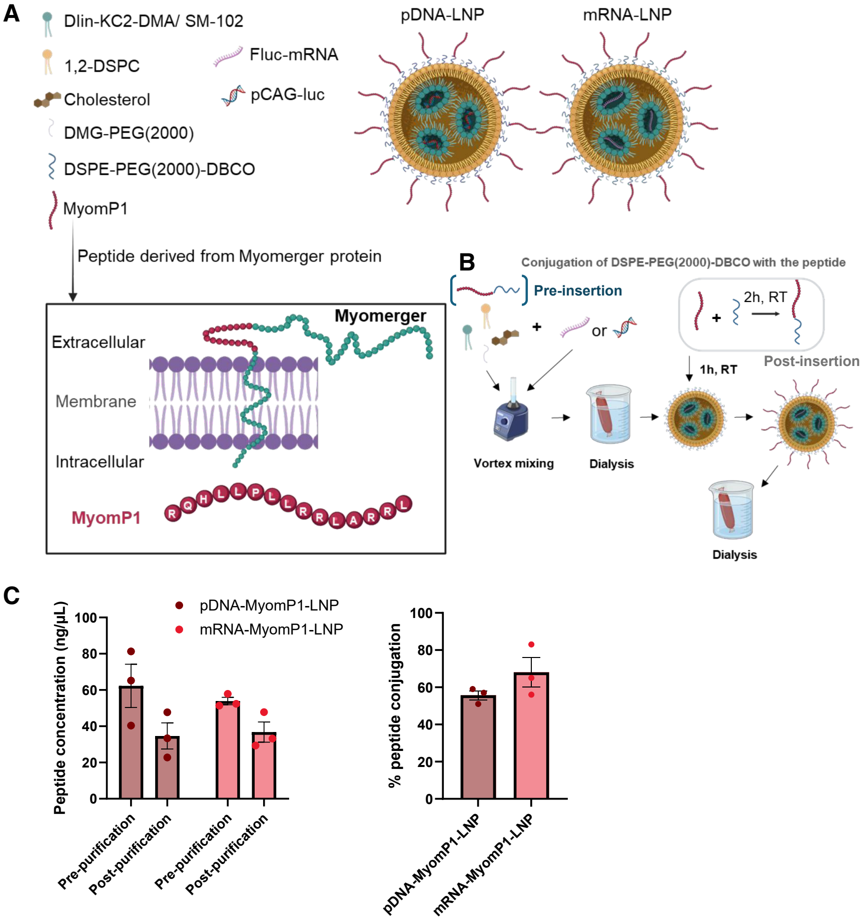

Figure 1. Formulation and surface modification of LNPs for muscle-targeted delivery

(A) Lipid and cargo composition of LNPs, and sequence and localization of the MyomP1 peptide in the Myomerger protein. The endogenous context of Myomerger in muscle cells is shown in the box. DLin-KC2-DMA was used for pDNA-LNPs, while SM-102 was used for mRNA-LNPs. (B) Schematic of the post-insertion strategy used to functionalize LNPs with the MyomP1 peptide. (C) Peptide conjugation assay. Peptide concentration (ng/μL) before and after LNP purification and the percentage of peptide conjugation were determined. $n = 4$; data are presented as mean ± SEM.

work has highlighted the use of muscle-specific fusogenic proteins to functionalize viral vectors and extracellular vesicles (EVs), considerably enhancing muscle transduction specificity.[28]

Altogether, developing a muscle-targeted nucleic acid delivery system remains challenging for next-generation gene therapies. In this study, we rationally designed a novel LNP functionalized with a peptide derived from the extracellular conserved region of Myomerger (MyomP1), a muscle-specific fusogenic protein, encapsulating either plasmid DNA (pDNA) or messenger RNA (mRNA). We assessed its transduction efficiency in both *in vitro* and *in vivo* models. Remarkably, our modified nonviral vector significantly reduced off-target liver transduction after local and systemic injections. These findings underscore the therapeutic potential of leveraging a minimal Myomerger-derived peptide to address key limitations of current gene delivery systems.

RESULTS

Validation of Myomerger-derived peptide-conjugated LNPs

LNPs encapsulating either pDNA or mRNA encoding the luciferase reporter protein were formulated, with their composition and schematic representation detailed in Figure 1A. We selected Dlin-KC2-DMA for pDNA-LNPs and SM-102 for mRNA-LNPs, as they are the most commonly used ionizable lipids for the corresponding LNP formulations.[18,24,29] To improve the skeletal muscle tropism of LNPs, we selected the MyomP1 peptide, derived from Myomerger, a muscle-specific fusogenic protein.[30] This peptide was selected for its ability to enhance interactions with muscle cell membranes and promote fusion, thereby facilitating cellular uptake and potential endosomal escape of the LNP cargo.[31] Its well-defined α-helical structure and high content of positively charged amino acids could further enhance LNP interactions with the negatively charged muscle cell membrane.[31,32] We conjugated MyomP1 to LNPs encapsulating luciferase pDNA (pCAG-luciferase, Addgene #55764) using both the post- and pre-insertion method (Figure 1B). The resulting formulation was then characterized by assessing nucleic acid encapsulation efficiency, particle size, polydispersity index (PDI), zeta potential, and peptide conjugation efficiency (Figures S1A–S1D).

The characterization revealed no significant differences between standard pDNA-LNPs and pDNA-MyomP1-LNPs (post-insertion) regarding encapsulation efficiency and PDI (Figures S1A and S1B). In contrast, pDNA-MyomP1-LNPs exhibited an increased particle size (107 ± 2 nm for MyomP1 vs. 96 ± 1 nm for pDNA-LNP) and zeta potential, consistent with the high content of positively charged amino acids in the MyomP1 peptide (Figures S1C and S1D). The pre-insertion strategy led to an approximately 50% reduction in encapsulation efficiency compared to post-insertion, accompanied by a marked increase in particle size to around 300 nm (Figures S1A–S1C). Consequently, all further experiments were conducted using the post-insertion approach. Approximately 50% of the initial peptide concentration was lost during LNP purification, suggesting either a reduced number of peptides decorating each LNP or a complete absence of peptides on some particles, resulting in a

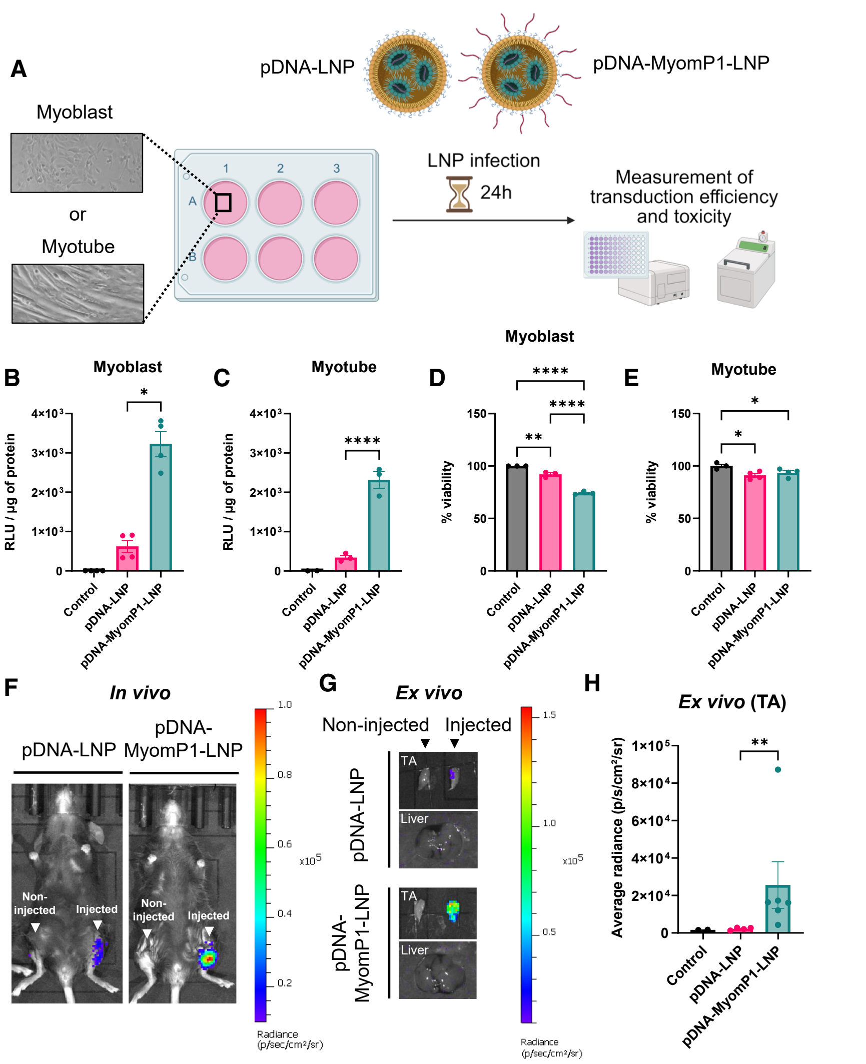

Figure 2. MyomP1-functionalized pDNA-LNPs enhance transduction efficiency *in vitro* and *in vivo*
Control corresponds to cells incubated with medium only or mice injected with PBS. LNP refers to lipid nanoparticles formulated with Dlin-KC2-DMA, DSPC, cholesterol, and DMG-PEG2000. MyomP1-LNP denotes LNPs conjugated with the MyomP1 peptide derived from the Myomerger protein. (A) *In vitro* study design: C2C12 myoblasts and myotubes were transduced with 2 μg and 4 μg of pDNA-LNPs per 1.2 × 10^6 cells, respectively. Transduction efficiency and cytotoxicity were assessed 24 h post-transduction. (B and C) Luciferase activity in myoblasts (B) and myotubes (C), shown as relative light units (RLUs) per μg of total protein. n = 3–4. (D and E) Cell viability in myoblasts (D) and myotubes (E) post-transduction. n = 3–4. (F) *In vivo* bioluminescence imaging of C57BL/6 mice injected with LNP or MyomP1-LNP. Injection was performed intramuscularly at a dose of 1 μg. Imaging was performed 24 h post-injection. Color scale indicates radiance (photons/s/cm^2/sr). (G) *Ex vivo* imaging of noninjected and injected TA and liver of C57BL/6 mice. Color scale indicates radiance (photons/s/cm^2/sr). (H) *Ex vivo* quantification of luminescence from TA muscles. n = 3–6; data are presented as mean ± SEM; $^{**}p < 0.01$. One-way ANOVA followed by Fisher's LSD test; $^{*}p < 0.05$, $^{**}p < 0.01$, $^{***}p < 0.001$, $^{****}p < 0.0001$.

subpopulation of partially or fully undecorated LNPs (Figure 1C). MyomP1-LNPs efficiently encapsulated both DNA and mRNA (Figure 1C). Morphological analysis using transmission electron microscopy (TEM) showed spherical particles with diameters close to 100 nm for both formulations. Notably, pDNA-MyomP1-LNPs displayed distinct lipid dot patterns on the particle surface (Figure S1E). These results validate the pDNA-MyomP1-LNP post-insertion formulation, which exhibits increased size and surface charge while maintaining high encapsulation efficiency and an effective peptide conjugation rate.

pDNA-MyomP1-LNPs improve transduction efficiency *in vitro* and *in vivo*

To evaluate the transduction efficiency of pDNA-MyomP1-LNPs in muscle cells, C2C12 myoblasts and myotubes were transduced with 1 μg of pDNA-LNP per 1.2E+6 cells for 24 h, followed by assessment of transduction efficiency and cytotoxicity (Figure 2A). Subsequently, 1 μg of pDNA-LNP was injected intramuscularly into wild-type (WT) C57BL/6 mice to assess *in vivo* transduction efficacy.

pDNA-MyomP1-LNPs yielded up to 5-fold and 7-fold higher relative luminescence units per μg of protein (RLU/μg) compared to unmodified LNPs in myoblasts and myotubes, respectively (Figures 2B and 2C). This improved efficacy was correlated with an 18% reduction in cell viability for myoblasts treated with pDNA-MyomP1-LNPs compared to LNPs (Figure 2D). Both LNP formulations induced mild toxicity in myotubes, although to a lesser extent (Figure 2E). Consistent with the *in vitro* findings, intramuscular injection of pDNA-MyomP1-LNPs into WT mice resulted in a 10-fold increase in the luminescence signal in the tibialis anterior (TA) muscle compared to pDNA-LNPs, which showed a barely detectable signal (Figures 2F and 2G). However, the overall luminescence remained low (approximately 2.5E+4 p/s/cm^2/sr for MyomP1-LNPs), highlighting the limited transduction efficiency of pDNA-loaded LNPs. In conclusion, although MyomP1 functionalization improves transduction efficiency both *in vitro* and *in vivo* over unmodified pDNA-LNPs, substituting pDNA cargo with mRNA may be necessary to achieve therapeutically relevant levels of protein expression. *Ex vivo* luminescence measurements showed that mRNA-MyomP1-LNPs produced a 27.7-fold higher signal in the TA muscle compared with pDNA-MyomP1-LNPs (Figure S1F).

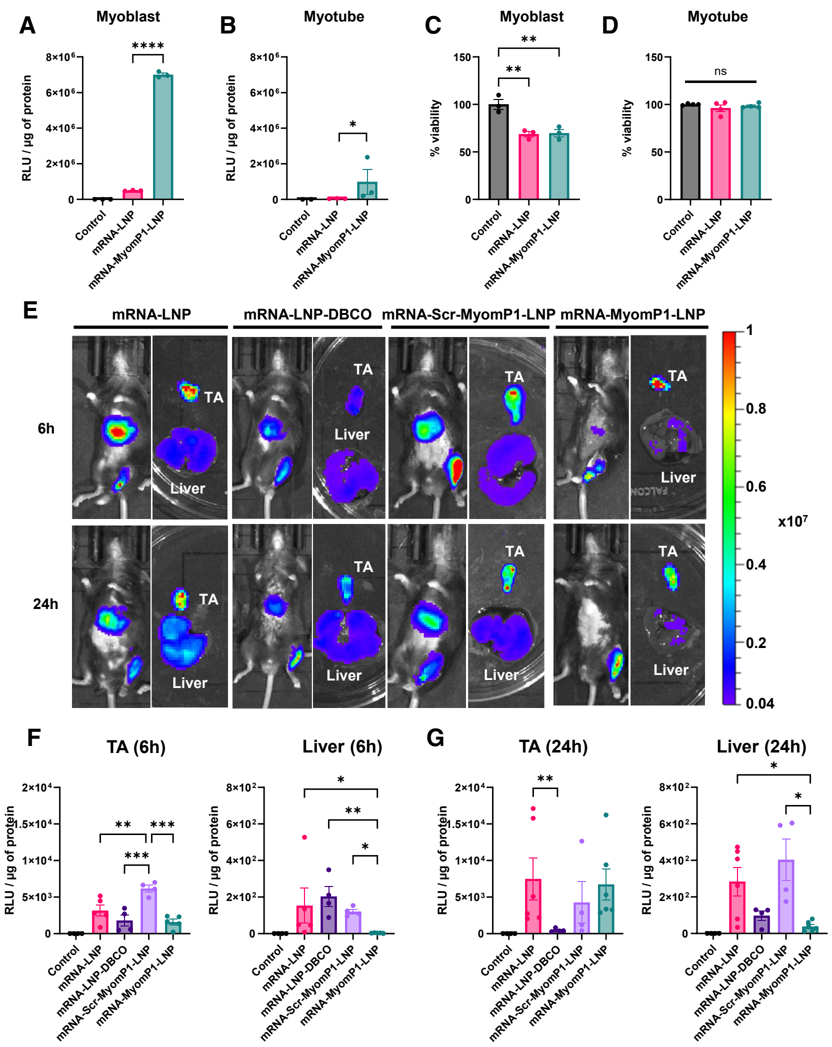

Figure 3. MyomP1-functionalized mRNA-LNPs enhance muscle targeting *in vitro* and reduce off-target transduction *in vivo*

Control corresponds to cells incubated with medium only or mice injected with PBS. LNP refers to lipid nanoparticles formulated with SM-102, DSPC, cholesterol, and DMG-PEG2000, whereas LNP-DBCO represents the same formulation incorporating DSPE-PEG(2000)-DBCO. MyomP1-LNP denotes LNPs conjugated with the MyomP1 peptide, while Scr-MyomP1-LNP refers to LNPs conjugated with a scrambled version of the MyomP1 peptide. (A and B) Luciferase activity in C2C12 myoblasts (A) and myotubes (B) after transduction with mRNA-LNPs. (C and D) Viability of myoblasts (C) and myotubes (D) post-transduction. $n = 3$. (E) *In vivo* and *ex vivo* bioluminescence imaging at 6 and 24 h post-injection of mRNA-LNPs in C57BL/6 mice. mRNA-LNPs were injected intramuscularly into the TA at a dose of 1 µg. Color scale indicates radiance (photons/s/cm^2/sr). (F and G) *Ex vivo* luciferase activity in TA and liver at 6 h (F) and 24 h (G), shown as RLU/µg of protein. $n = 4–5$. One-way ANOVA followed by Fisher's LSD test; *$p < 0.05$, **$p < 0.01$, ***$p < 0.001$, ****$p < 0.0001$.

Nevertheless, we cannot exclude the possibility that the integrity of the pDNA was altered during LNP formulation.

mRNA-MyomP1-LNPs enhance *in vitro* transduction efficiency and reduce off-target expression after local injection *in vivo*

To achieve better transduction efficacy, luciferase-encoding mRNA was used to replace pDNA as the cargo. Thus, mRNA-LNPs underwent a similar characterization process as pDNA-LNPs and exhibited similar variations between unmodified mRNA-LNPs and mRNA-MyomP1-LNPs (Figure 1C; Figures S2A–S2E). A dose of 1 µg of mRNA-LNP per 1.2E+6 cells *in vitro* and per TA muscle *in vivo* was applied for both cultured muscle cells and mice, respectively. For *in vivo* studies, mRNA-LNP-DBCO (LNPs containing DSPE-PEG(2000)-DBCO without peptide conjugation) and mRNA-Scr-MyomP1-LNPs (LNPs conjugated with a scrambled MyomP1 peptide maintaining the same amino acid composition and overall charge as MyomP1) were included as additional controls.

The morphology of mRNA-MyomP1-LNPs was visualized by cryo-TEM, and the results showed the presence of bleb structures in both standard and mRNA-MyomP1-LNPs (Figure S2E). This phenomenon was observed post-dialysis and is indicative of phase separation, with ionizable lipids and mRNA segregating on one side, and DSPC (Distearoylphosphatidylcholine) and cholesterol on the other.[33] The improvement in transduction efficiency was conserved with mRNA-MyomP1-LNPs in both myoblasts and myotubes (Figures 3A, 3B, and S3A–S3D). mRNA-MyomP1-LNPs yielded up to 14-fold and 22-fold higher relative luminescence units per µg of protein (RLU/µg) and 9-fold and 75-fold higher protein expression compared to mRNA-LNPs in myoblasts and myotubes, respectively. While a 30% reduction in cell viability was observed in murine myoblasts for both LNP formulations, no significant toxicity was detected in myotubes (Figures 3C and 3D). Notably, similar results were obtained in human myoblasts transfected with mRNA-MyomP1-LNPs, showing a 3.6-fold increase in luciferase activity and a 7-fold increase in protein expression compared to mRNA-LNPs (Figures S3A, S3B. and S4A–S4D). Additionally, mRNA-MyomP1-LNPs exhibited dose-dependent toxicity (Figure S3F). Over time, the mRNA-MyomP1-LNP formulation showed increased particle size and PDI, along with a progressive decrease in encapsulation from 2 to 4 weeks at 4°C (Figure S4). The superior efficacy of mRNA-MyomP1-LNPs over mRNA-LNPs in transducing muscle cells was not observed *in vivo* at either 6 or 24 h post-injection in TA muscles (Figures 3E–3H). *In vivo* imaging revealed a strong off-target luminescence signal in the liver following administration of

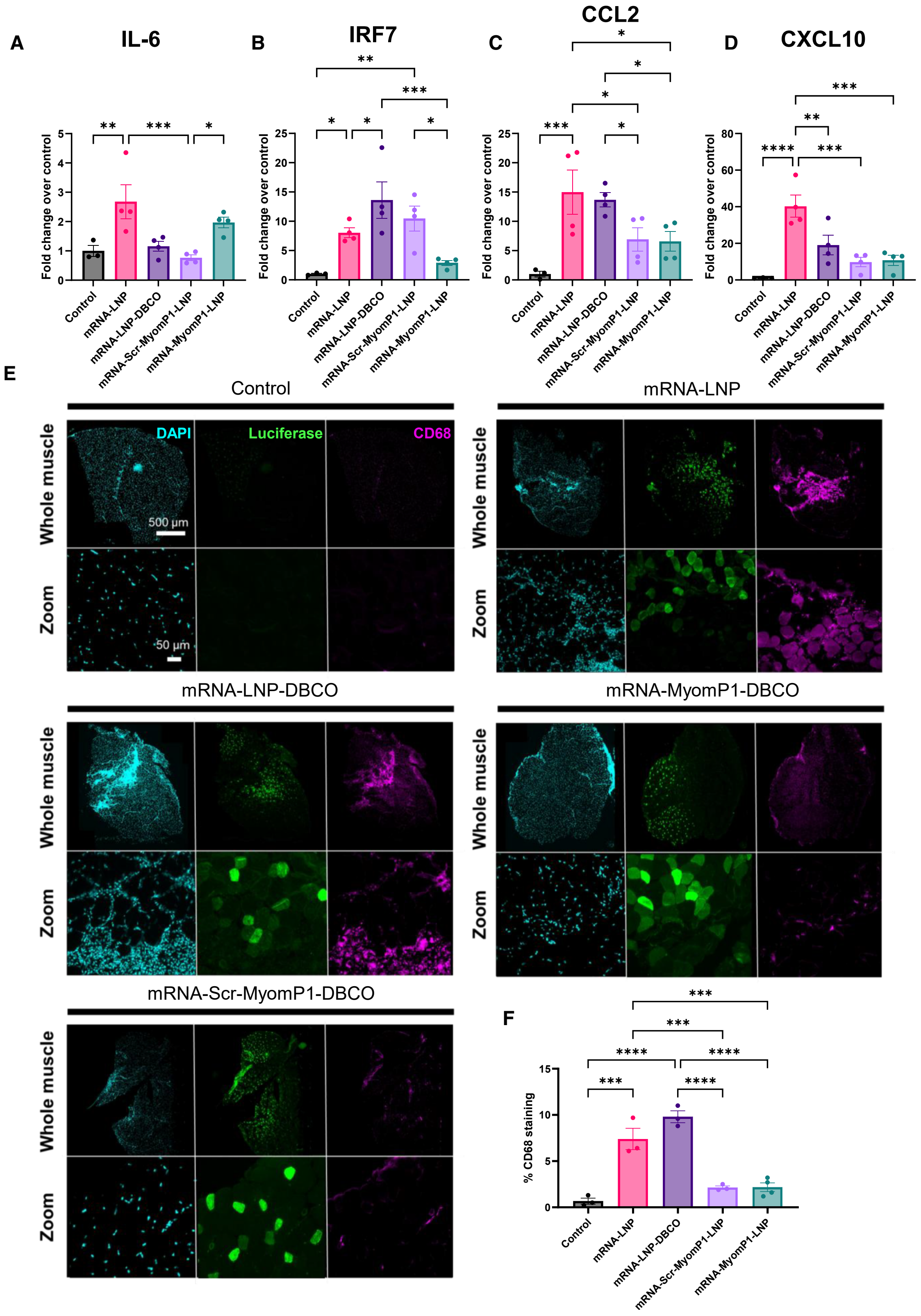

(legend on next page)

mRNA-LNPs compared to mRNA-MyomP1-LNPs, consistent with the known hepatic tropism of conventional LNPs. mRNA-MyomP1-LNPs showed a 51-fold and 7-fold decrease in liver signal at 6 and 24 h, respectively, compared with LNPs (Figures 3E–3G). Scr-MyomP1-LNP and MyomP1-LNP displayed similar transduction rates in the TA 24 h after injection. However, MyomP1-LNP liver transduction was lower than that of Scr-MyomP1-LNP at both 6 h and 24 h post-injection (Figures 3F and 3G).

Myomerger also contains a second extracellular helix, MyomP2, characterized by a highly hydrophobic sequence. mRNA-MyomP2-LNPs were tested in C2C12 myotubes and showed no improvement in transduction, unlike MyomP1-LNPs (Figures S2A–S2C). Notably, mRNA-MyomP2-LNPs exhibited no significant difference in luminescence in either the TA muscle or the liver compared to control mRNA-LNPs (Figures S2D and S2E).

To further investigate the mechanism underlying the liver de-targeting effect observed with MyomP1 functionalization, we evaluated the impact of recombinant ApoE protein on mRNA-LNP formulations (Figures S7A and S7B). The results showed that high ApoE concentrations increased the transduction efficiency of mRNA-LNP, mRNA-LNP-DBCO, and mRNA-Scr-MyomP1-LNP, whereas it remained unchanged for mRNA-MyomP1-LNP (Figures S7C–S7F).

These findings support that MyomP1 enhances muscle cell mRNA transduction *in vitro* and confers a liver de-targeting effect after local injection *in vivo* by limiting ApoE adsorption.

mRNA-MyomP1-LNPs reduce immunogenicity compared to standard LNPs *in vivo*

mRNA-LNPs are known to induce a strong immune response, including overactivation of innate immune pathways and macrophage recruitment.[21,22] To evaluate the impact of MyomP1 functionalization on the immune response, the expression levels of several immune-related genes were assessed in the inguinal lymph nodes, a site of mRNA-LNP drainage following intramuscular injection.[34,35] In parallel, macrophage infiltration at the injection site was analyzed by immunofluorescence under different conditions.

At 6 h post-injection, mRNA-LNPs induced a strong upregulation of innate immunity-associated genes, including interleukin-6 (IL-6), interferon regulatory factor 7 (IRF7), chemokine C-C motif ligand 2 (CCL2), and chemokine C-X-C motif ligand 10 (CXCL10) in lymph nodes (Figures 4A–4D). In contrast, mRNA-MyomP1-LNP led to a significant decrease in the expression of IRF7, CCL2, and CXCL10. Of note, functionalization of mRNA-LNP with Scr-MyomP1 also resulted in decreased RNA levels of CCL2 and CXCL10 (Figures 4C and 4D). In addition, immunofluorescence analysis revealed pronounced macrophage infiltration in TA muscles injected with mRNA-LNP and mRNA-LNP-DBCO, which was reduced in mRNA-MyomP1-LNP- and mRNA-Scr-MyomP1-LNP-injected muscles (Figures 4E and 4F). Overall, these results demonstrate reduced immunogenicity following functionalization of mRNA-LNP with MyomP1 and Scr-MyomP1.

mRNA-MyomP1-LNPs reduce liver expression after systemic injection and modulate adaptive immune response *in vivo*

To evaluate whether MyomP1-functionalized mRNA-LNPs retain their liver de-targeting capability following systemic administration, mRNA-LNPs were injected retro-orbitally into wild-type C57BL/6 mice at a dose of 3 μg. Bioluminescence imaging of several muscles and the liver was performed 24 h post-injection, followed by quantification of luciferase activity. In parallel, adaptive immune responses were assessed 1 week after systemic administration at the same dose to determine T and B cell distribution.

At 24 h post-injection, mice treated with mRNA-LNP or mRNA-LNP-DBCO exhibited strong bioluminescent signals in the liver, whereas mRNA-MyomP1-LNP-treated mice showed a lower hepatic signal (Figures 5A–5C). For whole-body analyses, unshaved abdominal hair may attenuate bioluminescent liver signals during IVIS (*In Vivo* Imagiging System) acquisition. Regarding muscle transduction, luminescence was detected in the TA, quadriceps, and gastrocnemius muscles of mice injected with mRNA-LNP or mRNA-MyomP1-LNP (Figure 5B). Luminescence was detected in the spleen in all groups (Figure 5B). Quantitative analysis of luciferase activity revealed that mRNA-LNP and mRNA-MyomP1-LNP presented 6.2- and 9.6-fold higher transduction in the gastrocnemius compared to mRNA-LNP-DBCO, respectively (Figure 5C). Conversely, mRNA-MyomP1-LNP showed 8.7- and 5.5-fold lower liver transduction compared to mRNA-LNP and mRNA-LNP-DBCO, respectively (Figure 5D). Overall, the gastrocnemius-to-liver luciferase activity ratio was significantly higher in mice receiving mRNA-MyomP1-LNP than in those receiving the control formulations (Figure 5E).

One week post-injection, splenocytes were analyzed to assess T and B cell populations as well as effector T cell subsets (Figure 6A). The

Figure 4. MyomP1-functionalized mRNA-LNPs reduce innate immune activation following intramuscular injection

Control corresponds to mice injected with PBS. LNP refers to lipid nanoparticles formulated with SM-102, DSPC, cholesterol, and DMG-PEG2000, whereas LNP-DBCO represents the same formulation incorporating DSPE-PEG(2000)-DBCO. MyomP1-LNP denotes LNPs conjugated with the MyomP1 peptide, while Scr-MyomP1-LNP refers to LNPs conjugated with a scrambled version of the MyomP1 peptide. Tissues were collected 6 h after mRNA-LNP injection. mRNA expression of IL-6 (A), IRF7 (B), CCL2 (C), and CXCL10 (D) was measured by RT-qPCR in C57BL/6 lymph node tissues and is presented as fold change relative to controls. n = 3–4. (E) Immunofluorescence of TA muscle sections from LNP-injected mice. Representative images of whole muscle sections and corresponding high-magnification views are shown. Scale bars are indicated. The zoomed region of the whole muscle is indicated by a white square. (F) Quantification of CD68-positive staining in TA muscle. CD68 is a macrophage-specific marker. The percentage of CD68 staining was determined by dividing the CD68-positive area by the total area of the muscle section. n = 3–4. One-way ANOVA followed by Fisher's LSD test; *p < 0.05, **p < 0.01, ***p < 0.001, ****p < 0.0001.

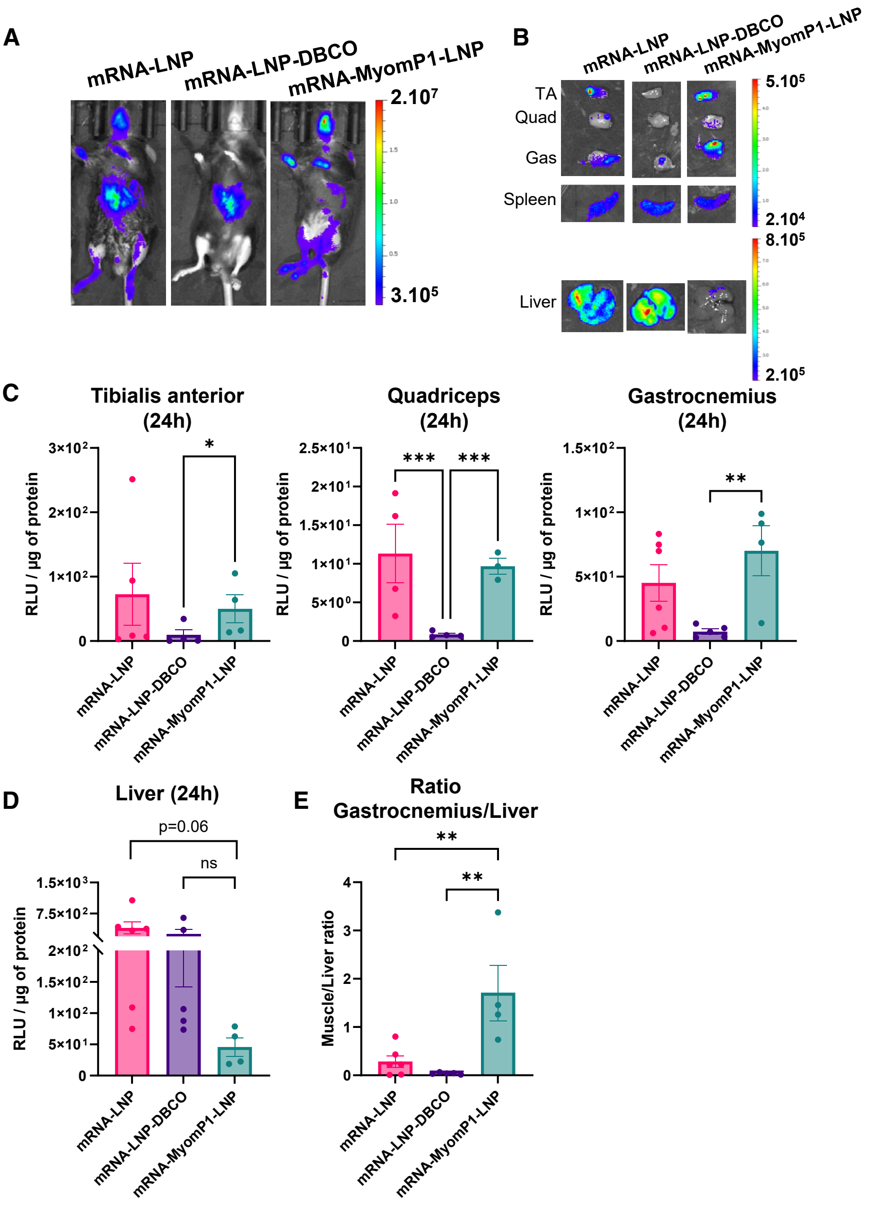

Figure 5. MyomP1-functionalized mRNA-LNPs reduce off-target transduction *in vivo* after intravenous injection

(A and B) *In vivo* (A) and *ex vivo* (B) bioluminescence imaging at 24 h post-injection of mRNA-LNPs in C57BL/6 mice. mRNA-LNPs were injected retro-orbitally at a dose of 3 μg. Color scale indicates radiance (photons/s/cm^2/sr). (C and D) Luciferase activity in tibialis anterior, quadriceps, and gastrocnemius muscles (C) and liver (D) at 24 h, shown as RLU/μg of protein. n = 4–6. (E) Ratio of luciferase activity in gastrocnemius muscle over liver. n = 4–6. One-way ANOVA followed by Fisher's LSD test or Kruskal-Wallis test followed by Dunn's LSD test; $^*p < 0.05$, $^{**}p < 0.01$, $^{***}p < 0.001$.

results showed an increased proportion of CD4$^+$ T cells in the mRNA-MyomP1-LNP group compared to mRNA-LNP, without a corresponding increase in effector CD4$^+$ T cells (Figures 6B and 6C). No significant changes were observed in CD8$^+$ T cell populations (Figure 6D). Notably, the proportion of B cells was reduced in the mRNA-MyomP1-LNP group compared to mRNA-LNP (Figure 6E).

Altogether, these findings demonstrate that MyomP1 functionalization confers a liver detargeting effect even after systemic administration and is associated with mild modulation of acquired immune cell populations.

DISCUSSION

Despite significant progress in gene therapy, current vectors for gene delivery still present several limitations, including immunogenicity and off-target transduction, particularly in the liver. In this study, we developed a muscle-targeting LNP system by functionalizing the LNP surface with MyomP1, a peptide derived from the extracellular domain of Myomerger, a muscle-specific fusogenic protein. Our strategy resulted in reduced liver tropism following intramuscular and intravenous delivery of mRNA-MyomP1-LNP in mice.

MyomP1 functionalization enhances *in vitro* transduction efficiency across different cargo types

Previous studies were conducted to assess the ability of LNPs to encapsulate different types of nucleic acids.[29,36] Here, we confirmed that MyomP1 significantly improves *in vitro* transduction efficiency in murine and human muscle cell lines, both at myoblast and differentiated myotube states, regardless of the nucleic acid cargo. This suggests that MyomP1 primarily facilitates cellular uptake or intracellular trafficking. Indeed, these results correlate with the study of Hindi et al., where lentiviruses or EVs decorated with the full-length Myomerger and Myomaker proteins exhibit enhanced cellular transduction.[28] However, while both pDNA- and mRNA-LNPs conjugated with MyomP1 showed increased transduction efficiency, pDNA-LNPs remained markedly less efficient, most likely due to the nuclear barrier limiting transgene protein expression. These results are consistent with previous findings showing that nuclear entry represents a bottleneck in pDNA delivery systems, in contrast to mRNA delivery, which only requires cytosolic translation.[37] Therefore, mRNA appears to be a more suitable cargo for LNP-based therapeutic applications in muscle cells.

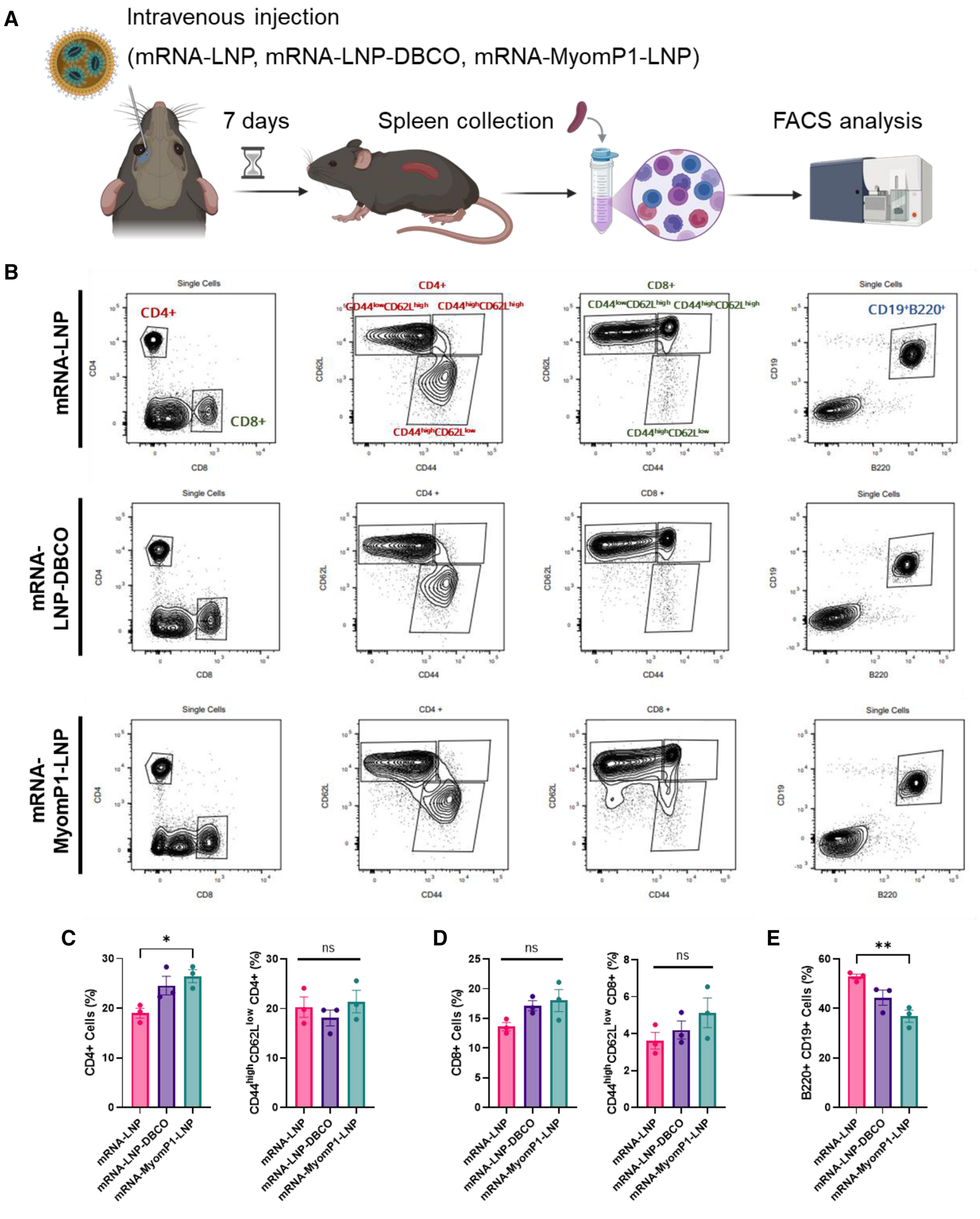

Figure 6. MyomP1-functionalized mRNA-LNPs modulate adaptive immune responses

All analyses were performed on splenocytes collected 7 days after systemic administration of mRNA-LNPs at a dose of 3 μg. (A) Schematic representation of the study design for evaluating adaptive immune cell populations. (B) Representative flow cytometry plots showing T and B cell populations under different conditions. Effector T cells ($CD4^+$ and $CD8^+$) are defined as $CD44^{high}CD62L^{low}$ cells. (C and D) Quantification of total $CD4^+$ (C) and $CD8^+$ (D) T cell populations, as well as their corresponding effector subsets ($CD44^{high}CD62L^{low}$). (E) Quantification of total B cell populations. $n = 3$; one-way ANOVA followed by Fisher's LSD test; $^*p < 0.05$; $^{**}p < 0.01$.

mRNA-MyomP1-LNPs reduce liver off-target and show muscle tropism *in vivo*

Off-target liver transduction remains a serious concern in AAV-based gene delivery for rare muscular diseases, leading to the death of several patients in clinical trials due to hepatotoxicity.[9–11] Liver accumulation of mRNA-LNPs is also a main bottleneck compromising tissue-specific delivery. Here, our studies revealed a strong liver signal in mice injected intramuscularly or systemically with standard mRNA-LNP and mRNA-LNP-DBCO, whereas mRNA-MyomP1-LNP substantially reduced hepatic expression 24 h after injection. These results confirm that MyomP1 functionalization promotes skeletal muscle tropism and liver detargeting of mRNA-LNPs following both local and systemic administration. They also suggest that MyomP1 confers a partial liver detargeting effect by reducing adsorption of mRNA-LNPs by ApoE lipoproteins.[20] However, it remains unclear whether mRNA-MyomP1-LNPs are still taken up by hepatic macrophages without transgene expression or whether the peptide actively inhibits hepatic uptake. mRNA-LNPs and mRNA-MyomP1-LNPs exhibited comparable levels of muscle transduction following both intramuscular and systemic administration. Although the overall signal appears lower than reported in previous studies, this may be due to the low dose (1 μg) used, which is approximately 7- to 12-fold lower than in previous reports.[24,33] For future proof-of-concept therapeutic studies, evaluating higher doses will be important to determine whether muscle transduction can be enhanced.

MyomP1 reduces local immune activation

mRNA-LNPs are known to trigger robust innate immune responses.[21,38] Here, a major advantage of mRNA-MyomP1-LNPs is the attenuation of immunogenicity. Indeed, mRNA-MyomP1-LNPs significantly reduced the expression of key inflammatory markers in draining lymph nodes and diminished macrophage recruitment at the injection site. These results raise the hypothesis that MyomP1 may mimic a self-antigen or interfere with pattern recognition receptor (PRR) engagement. This effect has been demonstrated with CD47-decorated nanoparticles, where macrophage-mediated clearance is strongly delayed by the presence of a "don't eat me" signal.[39] Nevertheless, some degree of activation was still observed with mRNA-MyomP1-LNPs, possibly due to incomplete peptide coverage on the mRNA-LNP surface, as suggested by the moderate conjugation efficiency of approximately 50% measured post-purification.

Conclusion

In conclusion, our study presents mRNA-MyomP1-LNPs as a promising nonviral vector platform for muscle-directed gene delivery. This approach addresses key limitations, including off-target liver expression and immune activation. Moreover, previous findings show enhanced transduction by Myomerger- and Myomaker-pseudotyped EVs in the DMD mouse model (mdx), suggesting that MyomP1 may similarly demonstrate improved efficacy in this disease model.[28] However, systemic administration remains a critical challenge. Further optimization of the mRNA-LNP composition—including ionizable lipid selection, particle size, and enhanced MyomP1 surface density—may be required to achieve efficient skeletal muscle transduction following systemic delivery.

MATERIALS AND METHODS

LNP synthesis

LNPs were formulated by vortex mixing for 20 s between an aqueous phase containing luciferase mRNA (Trilink, #L-7202) or pDNA (Addgene, #55764) in sodium acetate (50 mM, pH 4) and an organic phase containing the lipids (Dlin-KC2-DMA or SM-102, 1,2-DSPC, cholesterol, DMG-PEG2000 (1,2-dimyristoyl-rac-glycero-3-methoxypolyethylene glycol-2000)) at a 3:1 volume ratio, with an N/P ratio (ratio between the amine groups in ionizable lipids and the phosphate groups in DNA or mRNA) of 12. The composition of the lipid mix is detailed in Table S1. The resulting LNP solution was dialyzed for 2 h using a Slide-A-Lyzer device with a 20 kDa molecular weight cut-off (MWCO) (Thermo Fisher Scientific, #66005).

Peptide synthesis and quantification

Peptides were synthesized on an ABI 443A synthesizer using standard Fmoc chemistry with a Lys(N3)-OH (Iris Biotech, #FAA1394) attached to the N terminus and purified by reversed-phase liquid chromatography to a purity of at least 97%. The predicted peptide mass was confirmed by mass spectrometry. Peptide concentrations were determined based on their dry weight.

LNP functionalization

LNPs were functionalized with the peptide MyomP1 using either the post-insertion or pre-insertion strategy. For the post-insertion method, the MyomP1 peptide (RQHLLPLLRRLARRL), MyomP2 peptide (QDMREALLSCLLFVL), or Scr-MyomP1 peptide (APLHRQLRLRALLRL) was incubated in PBS with the lipid DSPE-PEG2000-DBCO for 2 h at room temperature at a molar ratio (DBCO: peptide) of 1:2 and a volume ratio of 1:1. The resulting product was incubated with either pDNA-LNP or mRNA-LNP for 1 h at room temperature or stored for up to 1 week at 4°C until use. For the pre-insertion strategy, LNPs consisting of SM-102, DSPC, cholesterol, DMG-PEG(2000), and DSPE-PEG(2000)-DBCO (molar ratio 50:10:38.5:1.4:0.1) were prepared and subsequently incubated with peptides for 2 h at room temperature to allow conjugation. The final functionalized-LNPs were dialyzed for 2 h (Slide-A-Lyzer 20 kDa, Thermo Fisher Scientific) or dialyzed and concentrated using Amicon Ultra Centrifugal Filters, 100 kDa MWCO (Merck, #UFC810008).

Peptide concentration

The concentration of peptide contained in the functionalized-LNPs after dialysis was determined using the FLuoProdige protein quantification assay kit (OzBiosciences, #FPRO200). Briefly, 50 μL of pDNA-LNPs, mRNA-LNPs, or BSA standards prepared in PBS were mixed with 50 μL of FluoProdige dye working solution. After a 15 min incubation at room temperature, fluorescence was

measured using a plate reader at an excitation/emission wavelength of 518/605 nm.

LNP characterization

Hydrodynamic diameter and zeta potential of pDNA- and mRNA-LNPs were measured using a Zetasizer Nano ZS (Malvern Instruments, Malvern, UK). Size measurements were performed at 25°C by dynamic light scattering (DLS) at a backscatter angle of 173° in disposable polystyrene cuvettes. Zeta potential was assessed by laser Doppler micro-electrophoresis using folded capillary cells (DTS1070). Samples were diluted 1:100 in ultrapure water. Each value represents the mean of at least three independent measurements. Data are reported as Z-average diameter, PDI, and zeta potential (mV), calculated using the Smoluchowski approximation.

To calculate the encapsulation efficiency of both pDNA- or mRNA-LNPs, the dsDNA Broad Range and RNA High Sensitivity Assay Kit (Thermo Fisher Scientific, #Q33265 and #Q32852, respectively) were used. LNPs were incubated with 0.5% Triton X-100 at 37°C for 15 min to release the encapsulated pDNA or mRNA. Encapsulation efficiency (%) was determined using the formula: ([total nucleic acid concentration – free nucleic acid concentration]/total nucleic acid concentration) × 100.

Cryo-TEM

Cryo-samples were prepared using a Vitrobot Mark IV (Thermo Fisher Scientific) set to 15°C with 95% humidity. 3 μL of the sample was applied onto a holey carbon grid covered with a 2 nm continuous carbon layer (Quantifoil R3.5/1 200 mesh), rendered hydrophilic by a 90-s treatment in a Fischione 1070 plasma cleaner operating at 34% power with a gas mixture of 80% argon and 20% oxygen. After waiting 15 s, grids were blotted for 3 s (blot force 5) and flash-frozen into precooled liquid ethane. Images were acquired on a Glacios (Thermo Fisher Scientific) microscope operating at 200 kV using the SerialEM software on a 4k × 4k Gatan K2 Summit direct electron detector.

Cell culture

C2C12 cells (supplied by EEAC, #91031101) and C25 cells (provided by Anne Bigot) were seeded at a density of 3E+5 cells per well in 6-well plates. When C2C12 myoblasts reached approximately 80% confluence, the growth medium (DMEM, Thermo Fisher Scientific, #31600-083, supplemented with 20% fetal calf serum, Sigma, #F7524, and gentamycine 40 μg/mL, Sigma) was replaced with differentiation medium (DMEM GLUTAMAX-I supplemented with 2% horse serum, Thermo Fisher Scientific, #41965039), which was changed daily until complete myotube differentiation. C2C12 myoblasts and myotubes, as well as C25 myoblasts, were transfected with pDNA-LNPs or mRNA-LNPs at a dose of 1 μg. Cells were harvested 24 h after transfection for further analysis.

ApoE binding assay

mRNA-LNP, mRNA-LNP-DBCO, mRNA-Scr-MyomP1-LNP, and mRNA-MyomP1-LNP formulations were each incubated with mouse ApoE lipoprotein (Acrobiosystems, #APE-M52H5) at different concentrations of 0, 1, and 5 μg/mL for 1 h at 37°C. Subsequently, C2C12 murine myoblasts (2.4E+5 cells per well, seeded in 24-well plates) were incubated with these solutions in serum-free DMEM (Thermo Fisher Scientific, #31600-083) for 24 h. Luciferase activity was then measured, and results (RLU/μg) were normalized as fold change relative to the control condition (no ApoE) for each mRNA-LNP formulation.

Luciferase activity assay

6 or 24 h after mRNA-LNP injection, animals were euthanized, and tissues (TA muscle and liver) were harvested. The collected organs were rapidly frozen in liquid nitrogen and subsequently stored at −80°C. Once frozen, the organs were pulverized into a fine powder, and 400 μL of lysis buffer (Promega #E4030, Wisconsin, US) was added. Samples were vortexed at room temperature for 15 min, followed by three cycles of freezing in liquid nitrogen and thawing in a 37°C water bath. The samples were then centrifuged for 3 min at 10,000 × g, and the resulting supernatant was retained for subsequent analysis. Luciferase activity was measured using a luminometer (Centro XS3 LB 960). Specifically, 100 μL of luciferase substrate (Promega #E4030, Wisconsin, US) was added to 20 μL of the extracted tissue supernatant, and relative light units (RLU) were measured 5 s after mixing. Luciferase activity was quantified by normalizing RLU to protein concentration (μg).

Animals

All mice were on a pure C57BL/6N background. They were housed in ventilated cages with free access to food and water, in temperature-controlled rooms under 12 h light/dark cycles. Eight-week-old mice were injected intramuscularly in the TA muscle with a dose of 1 μg for both mRNA- and pDNA-LNPs. In some cases, pDNA-LNP and pDNA-MyomP1-LNP were injected into different TAs of the same mouse. Animal care and experimentation were in accordance with French and European legislation and approved by the institutional ethics committee (project no. APAFIS# 53282-2024112510279287).

***In vivo* and *ex vivo* luminescence imaging**

For *in vivo* imaging, mice received a subcutaneous administration of D-luciferin in PBS at a dose of 150 mg/kg body weight. After 10 min, bioluminescence imaging was performed using an optical imaging system (IVIS Lumina XRMS). All images containing raw data were processed using Living Image software (PerkinElmer, MA, US). For *ex vivo* imaging, mice were dissected to collect the tissues of interest 10 min after D-luciferin injection (Medchem Express, #HY-12591B, 150 mg/kg), followed by bioluminescence imaging. The freehand tool was employed to extract total luciferase signals from either the whole body or specific organs. Data were exported in photons per second per square centimeter per steradian (p/sec/cm^2/sr) and represented as a pseudo-color overlay on a grayscale image of the animal.

Immunofluorescence

TA muscles from each injected mouse were collected, and 8-μm-thick muscle cross-sections were prepared for immunofluorescence

studies. Sections were fixed with 4% paraformaldehyde, permeabilized with 0.02% Triton X-100 in PBS, and washed with PBS 1X. Nonspecific binding was blocked for 1 h with 3% BSA in PBS. Primary antibodies—anti-CD68 (Bio-Rad, #MCA1957GA) and anti-firefly luciferase (Invitrogen, MA5-50410)—were diluted 1:200 to detect macrophages and transgene vector expression, respectively. Sections were incubated with primary antibodies overnight at 4°C in a humidified atmosphere. After a series of washings with PBS 1X, secondary antibodies Alexa Fluor 488 goat anti-rabbit and Alexa Fluor 647 goat anti-rat (dilution 1:400) were applied for 1 h at room temperature. After a series of washings, sections were mounted with ProLongTM Gold antifade reagent (Invitrogen #P36934, MA, US) and air-dried before imaging using a microscope (Zeiss, Germany).

RNA extraction and RT-qPCR

Frozen tissue samples were mechanically lysed using a Precellys homogenizer (Bertin Technologies, France) in TRIzol reagent (Thermo Fischer Scientific #15596026, MA, US) and processed according to the manufacturer's instructions to obtain total RNA. RNA concentration was determined by spectrophotometry (Nanodrop 2000, Thermo Fisher Scientific, MA, US). cDNA was synthesized from RNA using SuperScript IV Reverse Transcriptase (Thermo Fisher Scientific #18090010, MA, US). For RNA expression analysis, a 12 μL qPCR reaction was performed containing cDNA, SYBR Green Master Mix I (Roche #04707516001, Switzerland), primers for the gene of interest (*Il-6*, *Irf7*, *Ccl2*, *Cxcl10*), and the housekeeping gene (*Rps11*) primers (Table S2). Relative RNA expression for each condition was normalized to the housekeeping gene, and the results were expressed as fold change relative to the control group.

Western blot

Protein concentration was quantified using the PierceTM BCA Protein Assay (Thermo Fisher Scientific). 15 μg of protein in 4× Lane Marker Reducing Buffer (Thermo Fisher Scientific) were separated on 4%–15% Mini-PROTEAN TGX Precast Protein Gels (Bio-Rad) alongside the PageRuler Plus Prestained Protein Ladder (Thermo Fisher Scientific #26620). The gel was transferred to a nitrocellulose membrane (Bio-Rad) using a Transblot Turbo RTA transfer kit (Bio-Rad) for 10 min at 2.5 A. Protein loading was normalized based on GAPDH expression. Membranes were blocked for 1 h in tris-buffered saline (TBS)containing 5% nonfat dry milk and 0.1% Tween 20 (Merck), followed by incubation with antifirefly luciferase antibodies (Invitrogen, MA5-50410) diluted 1:1000 for 1 h at room temperature or overnight at 4°C. Subsequently, membranes were incubated with secondary antibodies coupled to horseradish peroxidase (Jackson ImmunoResearch). Membranes were visualized using an Amersham Imager 600 (GE Healthcare Life Sciences) after the addition of SuperSignal West Pico PLUS chemiluminescent substrate (Thermo Fisher Scientific). The Fiji software was used for the quantification of the band intensity in the images.

Flow cytometry analysis

Mice were sacrificed at 8 weeks of age, and spleens were immediately collected into 1.5 mL of ice-cold PBS containing 0.5% BSA and 2 mM EDTA (wash buffer [WB]). Cell suspensions were obtained by dissociating the spleens with a plunger in petri dishes. Splenocytes are filtered through a 50 μm nylon mesh, washed once with ice-cold WB, and centrifuged for 5 min at 1200 rpm at 4°C. The supernatant was aspirated, and red blood cells were lysed with 1 volume of 0.83% NH4Cl (0.7 mL per organ) for 5 min at 4°C. The lysis was stopped by adding 10 volumes of ice-cold WB, and cells were centrifuged. A second lysis was performed, followed by two additional washes with ice-cold WB. Cells were then counted and resuspended in ice-cold WB at 5E+7 cells/mL.

A total of 1E+7 splenocytes per condition were stained in 96-well U-bottom plates. All antibody dilutions and washes were performed in ice-cold WB. Cells were incubated for 30 min on ice with the antibody panel described in Table S3, followed by two washes with 200 μL of WB (centrifugation at 1200 rpm for 2 min at 4°C). Pellets were resuspended in 200 μL of WB before acquisition. DAPI (4′,6-diamidino-2-phenylindole) (1 μL/mL) was added immediately before analysis to exclude nonviable cells.

Flow cytometry analyses were performed immediately after staining on a BD LSRFortessa X-20 cytometer (BD Biosciences) using FACSDiva software with automatic compensation. Compensation was determined using single-color controls prepared with UltraComp eBeads Compensation Beads (Thermo Fisher Scientific, #01-2222-41). A minimum of 20,000 $CD4^{+}CD44^{high}CD62L^{low}$ events were acquired per sample. Data were analyzed using FlowJo software (Tree Star, v.10). Populations analyzed included total B cells ($CD19^{+}/B220^{+}$) and effector memory T cells ($CD44^{high}CD62L^{low}$). All raw data were archived on an internal server for reanalysis, and final gated populations were exported in PDF and JPEG formats.

Statistics

Data are presented as mean ± SEM. Statistical analyses were performed using GraphPad Prism software (v.10.0.2). Data were analyzed by an unpaired one-way ANOVA with Fisher's least significant difference test after verification of normal or log-normal distribution using the Shapiro-Wilk test. Otherwise, the Kruskal-Wallis test was used. All cell culture experiments were conducted in triplicate across three independent experiments. A p value of less than 0.05 was considered statistically significant.

DATA AVAILABILITY

All data are available upon request from the corresponding authors.

ACKNOWLEDGMENTS

We thank the Institut Clinique de la Souris for animal care, the Center of Integrated Biology, the IGBMC platforms (microscopy, proteomic, and electron microscopy), and the Integrated Structural Biology platform of the Strasbourg Instruct-ERIC center IGBMC-CBI, supported by FRISBI (ANR-10-INBS-0005). This work was supported by the Interdisciplinary Thematic Institute IMCBio+, as part of the ITI 2021–2028 program of the University of Strasbourg, CNRS, and Inserm, IdEx Unistra (ANR-10-IDEX-0002), the SFRI-STRAT'US project (ANR-20-SFRI-0012), and EUR IMCBio (ANR-17-EURE-0023) under the framework of the France 2030 Program, as well as by ANR Dynather (ANR-18-CE17-0006-02). We acknowledge Dr. Mayeul Collot for

his helpful guidance on peptide-LNP conjugation. We also thank the flow cytometry platform, especially Cécile Macquin and Muriel Philipps.

AUTHOR CONTRIBUTIONS

J.J. and J.L. designed and supervised the study. J.J. performed the *in vivo* and molecular experiments. J.J. and E.L. performed the *in vitro* experiments. C.C. conducted the electron microscopy. P.E. synthesized the peptide. N.A. assisted with the characterization of LNPs. J.J. wrote the manuscript. J.L. and N.A. edited the manuscript.

DECLARATION OF INTERESTS

The authors declare no competing interests.

SUPPLEMENTAL INFORMATION

Supplemental information can be found online at https://doi.org/10.1016/j.omtn.2025.102785.

REFERENCES

1. Rittiner, J., Cumaran, M., Malhotra, S., and Kantor, B. (2022). Therapeutic modulation of gene expression in the disease state: Treatment strategies and approaches for the development of next-generation of the epigenetic drugs. Front. Bioeng. Biotechnol. *10*, 1035543. https://doi.org/10.3389/fbioe.2022.1035543.
2. Naso, M.F., Tomkowicz, B., Perry, W.L., and Strohl, W.R. (2017). Adeno-Associated Virus (AAV) as a Vector for Gene Therapy. BioDrugs *31*, 317–334. https://doi.org/10.1007/s40259-017-0234-5.
3. Fang, C.-C., Wu, C.-F., Liao, Y.-J., Huang, S.-F., Chen, M., and Chen, Y.-M.A. (2018). AAV serotype 8-mediated liver specific GNMT expression delays progression of hepatocellular carcinoma and prevents carbon tetrachloride-induced liver damage. Sci. Rep. *8*, 13802. https://doi.org/10.1038/s41598-018-30800-3.
4. Manno, C.S., Chew, A.J., Hutchison, S., Larson, P.J., Herzog, R.W., Arruda, V.R., Tai, S.J., Ragni, M.V., Thompson, A., Ozelo, M., et al. (2003). AAV-mediated factor IX gene transfer to skeletal muscle in patients with severe hemophilia B. Blood *101*, 2963–2972. https://doi.org/10.1182/blood-2002-10-3296.
5. Mendell, J.R., Muntoni, F., McDonald, C.M., Mercuri, E.M., Ciafaloni, E., Komaki, H., Leon-Astudillo, C., Nascimento, A., Proud, C., Schara-Schmidt, U., et al. (2025). AAV gene therapy for Duchenne muscular dystrophy: the EMBARK phase 3 randomized trial. Nat. Med. *31*, 332–341. https://doi.org/10.1038/s41591-024-03304-z.
6. Buchlis, G., Podsakoff, G.M., Radu, A., Hawk, S.M., Flake, A.W., Mingozzi, F., and High, K.A. (2012). Factor IX expression in skeletal muscle of a severe hemophilia B patient 10 years after AAV-mediated gene transfer. Blood *119*, 3038–3041. https://doi.org/10.1182/blood-2011-09-382317.
7. Le Guiner, C., Servais, L., Montus, M., Larcher, T., Fraysse, B., Moullec, S., Allais, M., François, V., Dutilleul, M., Malerba, A., et al. (2017). Long-term microdystrophin gene therapy is effective in a canine model of Duchenne muscular dystrophy. Nat. Commun. *8*, 16105. https://doi.org/10.1038/ncomms16105.
8. Hoy, S.M. (2023). Delandistrogene Moxeparvovec: First Approval. Drugs *83*, 1323–1329. https://doi.org/10.1007/s40265-023-01929-x.
9. (2020). High-dose AAV gene therapy deaths. Nat. Biotechnol. *38*, 910. https://doi.org/10.1038/s41587-020-0642-9.
10. Morales, L., Gambhir, Y., Bennett, J., and Stedman, H.H. (2020). Broader Implications of Progressive Liver Dysfunction and Lethal Sepsis in Two Boys following Systemic High-Dose AAV. Mol. Ther. *28*, 1753–1755. https://doi.org/10.1016/j.ymthe.2020.07.009.
11. Chand, D., Mohr, F., McMillan, H., Tukov, F.F., Montgomery, K., Kleyn, A., Sun, R., Tauscher-Wisniewski, S., Kaufmann, P., and Kullak-Ublick, G. (2021). Hepatotoxicity following administration of onasemnogene abeparvovec (AVXS-101) for the treatment of spinal muscular atrophy. J. Hepatol. *74*, 560–566. https://doi.org/10.1016/j.jhep.2020.11.001.
12. Wu, Z., Yang, H., and Colosi, P. (2010). Effect of Genome Size on AAV Vector Packaging. Mol. Ther. *18*, 80–86. https://doi.org/10.1038/mt.2009.255.
13. Hamilton, H., Gomos, J., Berns, K.I., and Falck-Pedersen, E. (2004). Adeno-Associated Virus Site-Specific Integration and AAVS1 Disruption. J. Virol. *78*, 7874–7882. https://doi.org/10.1128/JVI.78.15.7874-7882.2004.
14. Shieh, P., Kuntz, N., Dowling, J., Müller-Felber, W., Bönnemann, C., Foley, D., Seferian, A., Servais, L., Lawlor, M., Noursalehi, M., et al. (2022). OP018: ASPIRO gene therapy trial in X-Linked Myotubular Myopathy (XLMTM): Update on preliminary efficacy and safety findings. Genet. Med. *24*, S350. https://doi.org/10.1016/j.gim.2022.01.568.
15. Hou, X., Zaks, T., Langer, R., and Dong, Y. (2021). Lipid nanoparticles for mRNA delivery. Nat. Rev. Mater. *6*, 1078–1094. https://doi.org/10.1038/s41578-021-00358-0.
16. Godbout, K., and Tremblay, J.P. (2022). Delivery of RNAs to Specific Organs by Lipid Nanoparticles for Gene Therapy. Pharmaceutics *14*, 2129. https://doi.org/10.3390/pharmaceutics14102129.
17. Hald Albertsen, C., Kulkarni, J.A., Witzigmann, D., Lind, M., Petersson, K., and Simonsen, J.B. (2022). The role of lipid components in lipid nanoparticles for vaccines and gene therapy. Adv. Drug Deliv. Rev. *188*, 114416. https://doi.org/10.1016/j.addr.2022.114416.
18. Zhang, L., More, K.R., Ojha, A., Jackson, C.B., Quinlan, B.D., Li, H., He, W., Farzan, M., Pardi, N., and Choe, H. (2023). Effect of mRNA-LNP components of two globally-marketed COVID-19 vaccines on efficacy and stability. npj Vaccines *8*, 156. https://doi.org/10.1038/s41541-023-00751-6.
19. Akinc, A., Maier, M.A., Manoharan, M., Fitzgerald, K., Jayaraman, M., Barros, S., Ansell, S., Du, X., Hope, M.J., Madden, T.D., et al. (2019). The Onpattro story and the clinical translation of nanomedicines containing nucleic acid-based drugs. Nat. Nanotechnol. *14*, 1084–1087. https://doi.org/10.1038/s41565-019-0591-y.
20. Sebastiani, F., Yanez Arteta, M., Lerche, M., Porcar, L., Lang, C., Bragg, R.A., Elmore, C.S., Krishnamurthy, V.R., Russell, R.A., Darwish, T., et al. (2021). Apolipoprotein E Binding Drives Structural and Compositional Rearrangement of mRNA-Containing Lipid Nanoparticles. ACS Nano *15*, 6709–6722. https://doi.org/10.1021/acsnano.0c10064.
21. Nelson, J., Sorensen, E.W., Mintri, S., Rabideau, A.E., Zheng, W., Besin, G., Khatwani, N., Su, S.V., Miracco, E.J., Issa, W.J., et al. (2020). Impact of mRNA chemistry and manufacturing process on innate immune activation. Sci. Adv. *6*, eaaz6893. https://doi.org/10.1126/sciadv.aaz6893.
22. Zelkoski, A.E., Lu, Z., Sukumar, G., Dalgard, C., Said, H., Alameh, M.-G., Mitre, E., and Malloy, A.M.W. (2025). Ionizable lipid nanoparticles of mRNA vaccines elicit NF-κB and IRF responses through toll-like receptor 4. npj Vaccines *10*, 73. https://doi.org/10.1038/s41541-025-01124-x.
23. Tursi, N.J., Tiwari, S., Bedanova, N., Kannan, T., Parzych, E., Okba, N., Liaw, K., Sárközy, A., Livingston, C., Trullen, M.I., et al. (2025). Modulation of lipid nanoparticle-formulated plasmid DNA drives innate immune activation promoting adaptive immunity. Cell Rep. Med. *6*, 102035. https://doi.org/10.1016/j.xcrm.2025.102035.
24. Chen, J., Xu, Y., Zhou, M., Xu, S., Varley, A.J., Golubovic, A., Lu, R.X.Z., Wang, K.C., Yeganeh, M., Vosoughi, D., and Li, B. (2023). Combinatorial design of ionizable lipid nanoparticles for muscle-selective mRNA delivery with minimized off-target effects. Proc. Natl. Acad. Sci. USA *120*, e2309472120. https://doi.org/10.1073/pnas.2309472120.
25. Dilliard, S.A., and Siegwart, D.J. (2023). Passive, active and endogenous organ-targeted lipid and polymer nanoparticles for delivery of genetic drugs. Nat. Rev. Mater. *8*, 282–300. https://doi.org/10.1038/s41578-022-00529-7.
26. Huang, D., Yue, F., Qiu, J., Deng, M., and Kuang, S. (2020). Polymeric nanoparticles functionalized with muscle-homing peptides for targeted delivery of phosphatase and tensin homolog inhibitor to skeletal muscle. Acta Biomater. *118*, 196–206. https://doi.org/10.1016/j.actbio.2020.10.009.
27. Wang, Y., Ma, Z., Jiang, L., Bojan, N., Sha, Y., Huang, B., Ming, L., Shen, J., and Pang, W. (2025). Specific muscle targeted delivery of miR-130a loaded lipid nanoparticles: a novel approach to inhibit lipid accumulation in skeletal muscle and obesity. J. Nanobiotechnol. *23*, 159. https://doi.org/10.1186/s12951-025-03225-0.
28. Hindi, S.M., Petrany, M.J., Greenfeld, E., Focke, L.C., Cramer, A.A.W., Whitt, M.A., Khairallah, R.J., Ward, C.W., Chamberlain, J.S., Podbilewicz, B., et al. (2023). Enveloped viruses pseudotyped with mammalian myogenic cell fusogens target skeletal muscle for gene delivery. Cell *186*, 2062–2077.e17. https://doi.org/10.1016/j.cell.2023.03.033.
29. Algarni, A., Pilkington, E.H., Suys, E.J.A., Al-Wassiti, H., Pouton, C.W., and Truong, N.P. (2022). *In vivo* delivery of plasmid DNA by lipid nanoparticles: the influence of

ionizable cationic lipids on organ-selective gene expression. Biomater. Sci. *10*, 2940–2952. https://doi.org/10.1039/D2BM00168C.

30. Quinn, M.E., Goh, Q., Kurosaka, M., Gamage, D.G., Petrany, M.J., Prasad, V., and Millay, D.P. (2017). Myomerger induces fusion of non-fusogenic cells and is required for skeletal muscle development. Nat. Commun. *8*, 15665. https://doi.org/10.1038/ncomms15665.

31. Gamage, D.G., Melikov, K., Munoz-Tello, P., Wherley, T.J., Focke, L.C., Leikina, E., Huffman, E., Diao, J., Kojetin, D.J., Prasad, V., et al. (2022). Phosphatidylserine orchestrates Myomerger membrane insertions to drive myoblast fusion. Proc. Natl. Acad. Sci. USA *119*, e2202490119. https://doi.org/10.1073/pnas.2202490119.

32. Chen, B., You, W., Wang, Y., and Shan, T. (2020). The regulatory role of Myomaker and Myomixer–Myomerger–Minion in muscle development and regeneration. Cell. Mol. Life Sci. *77*, 1551–1569. https://doi.org/10.1007/s00018-019-03341-9.

33. Cheng, M.H.Y., Leung, J., Zhang, Y., Strong, C., Basha, G., Momeni, A., Chen, Y., Jan, E., Abdolahzadeh, A., Wang, X., et al. (2023). Induction of Bleb Structures in Lipid Nanoparticle Formulations of mRNA Leads to Improved Transfection Potency. Adv. Mater. *35*, 2303370. https://doi.org/10.1002/adma.202303370.

34. Takanashi, A., Pouton, C.W., and Al-Wassiti, H. (2023). Delivery and Expression of mRNA in the Secondary Lymphoid Organs Drive Immune Responses to Lipid Nanoparticle-mRNA Vaccines after Intramuscular Injection. Mol. Pharm. *20*, 3876–3885. https://doi.org/10.1021/acs.molpharmaceut.2c01024.

35. Buckley, M., Araínga, M., Maiorino, L., Pires, I.S., Kim, B.J., Michaels, K.K., Dye, J., Qureshi, K., Zhang, Y.J., Mak, H., et al. (2025). Visualizing lipid nanoparticle trafficking for mRNA vaccine delivery in non-human primates. Mol. Ther. *33*, 1105–1117. https://doi.org/10.1016/j.ymthe.2025.01.008.

36. Pardi, N., Tuyishime, S., Muramatsu, H., Kariko, K., Mui, B.L., Tam, Y.K., Madden, T.D., Hope, M.J., and Weissman, D. (2015). Expression kinetics of nucleoside-modified mRNA delivered in lipid nanoparticles to mice by various routes. J. Control. Release *217*, 345–351. https://doi.org/10.1016/j.jconrel.2015.08.007.

37. Cavegn, A., Waldner, S., Wang, D., Sedzicki, J., Kuzucu, E.Ü., Zogg, M., Lotter, C., and Huwyler, J. (2025). Intracellular processing of DNA-lipid nanoparticles: A quantitative assessment by image segmentation. J. Control. Release *382*, 113709. https://doi.org/10.1016/j.jconrel.2025.113709.

38. Ndeupen, S., Qin, Z., Jacobsen, S., Bouteau, A., Estanbouli, H., and Igyártó, B.Z. (2021). The mRNA-LNP platform's lipid nanoparticle component used in preclinical vaccine studies is highly inflammatory. iScience *24*, 103479. https://doi.org/10.1016/j.isci.2021.103479.

39. Rodriguez, P.L., Harada, T., Christian, D.A., Pantano, D.A., Tsai, R.K., and Discher, D.E. (2013). Minimal "Self" Peptides That Inhibit Phagocytic Clearance and Enhance Delivery of Nanoparticles. Science *339*, 971–975. https://doi.org/10.1126/science.1229568.

OMTN, Volume *37*

Supplemental information

Myomerger-derived peptide enhances skeletal muscle tropism and reduces liver transduction of lipid nanoparticles for gene delivery

Jacqueline Ji, Eva Lipkow, Nicolas Anton, Corinne Crucifix, Pascal Eberling, and Jocelyn Laporte

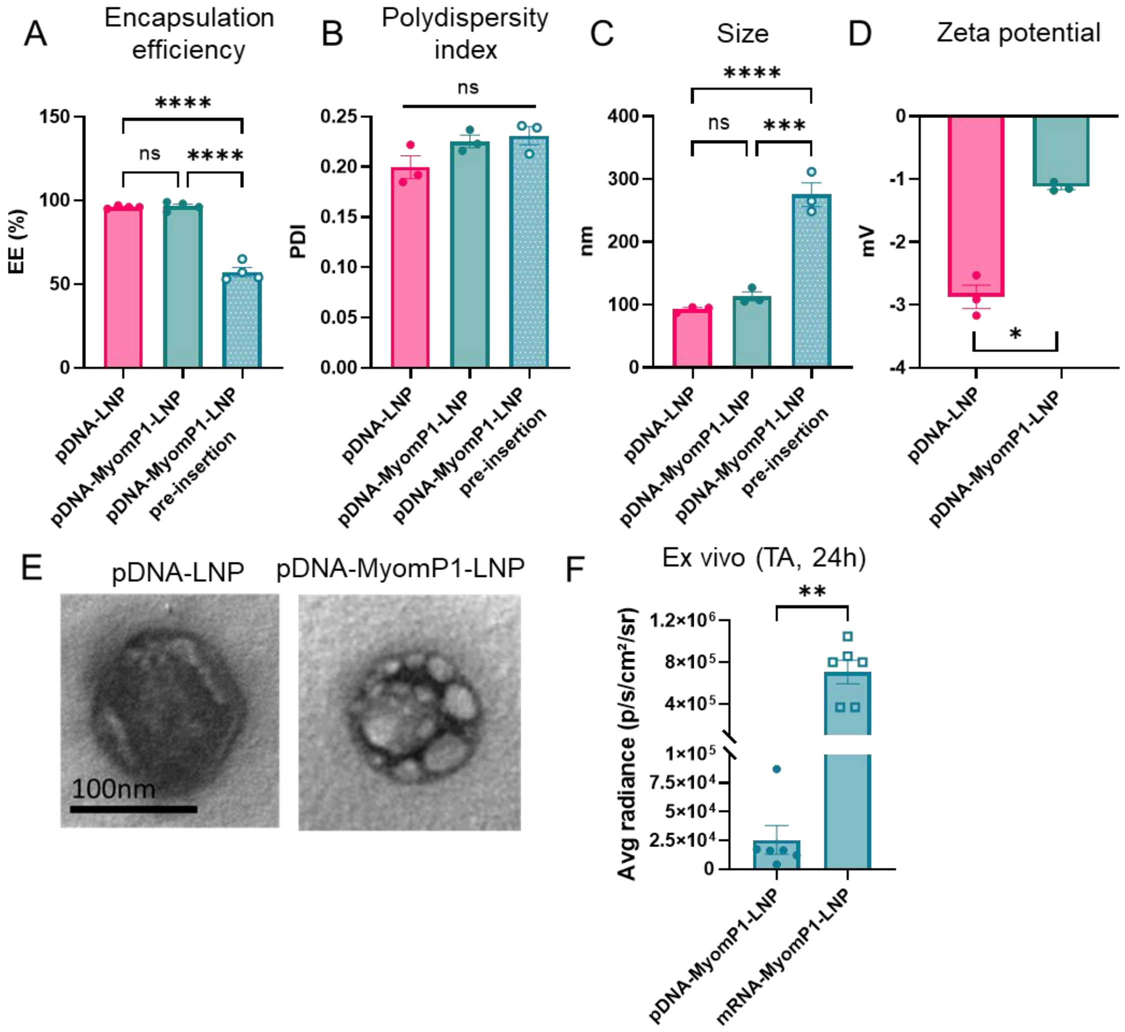

Figure S1. Characterization of peptide-functionalized pDNA-LNPs. pDNA-MyomP1-LNP refers to the LNP formulation in which DSPE-PEG(2000)-DBCO/MyomP1 is incorporated after LNP formation (post-insertion). In contrast, pDNA-MyomP1-LNP with pre-insertion indicates that MyomP1 is incorporated by incubating it with LNPs that already contain DBCO during the formulation process. **(A)** Encapsulation efficiency of pDNA-LNPs. Data are presented as mean ± SEM; ****p<0.0001. **(B)** Polydispersity index of pDNA-LNPs. Data is presented as the mean ± SEM. **(C)** Size of pDNA-LNPs. Data are presented as mean ± SEM; ***p<0.001, ****p<0.0001. **(D)** Zeta potential of pDNA-LNPs. The zeta potential was measured at pH=7 in PBS buffer. Data are presented as mean ± SEM; *p<0.05. **(E)**

Transmission electron microscopy images of pDNA-LNPs. Scale bar is indicated. (**F**) Ex vivo quantification of bioluminescence from TA muscles 24h after pDNA-MyomP1-LNP or mRNA-MyomP1-LNP injection. n= 6; Data are presented as mean ± SEM; **p<0.01.

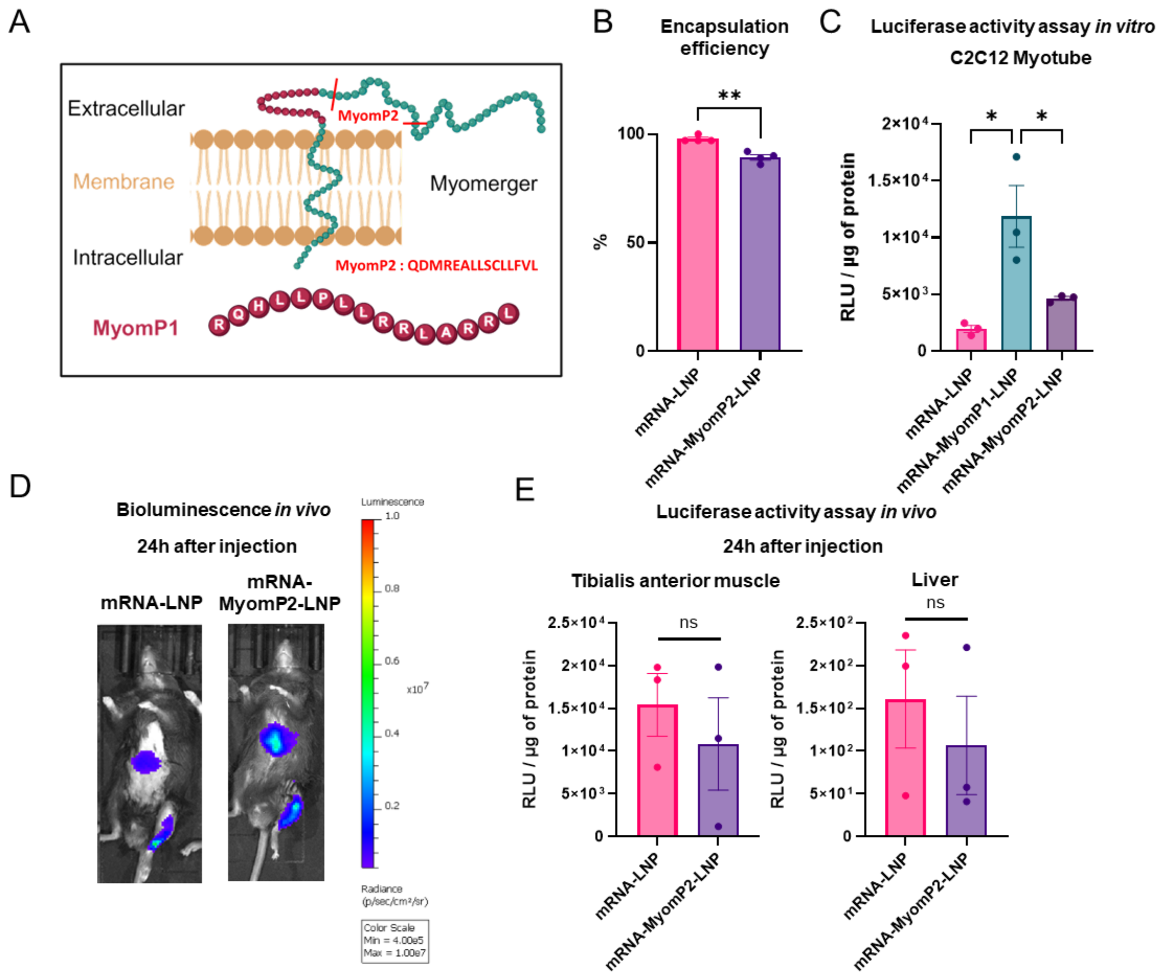

Figure S2. **MyomP2 functionalization shows no enhanced transduction efficiency or off-target effects *in vitro* and *in vivo*.** (**A**) Illustration of the peptide MyomP2 compared to MyomP1. Sequence are indicated in blue and red respectively. (**B**) Encapsulation efficiency. (**C**) Luciferase activity in C2C12 myotubes after transduction with mRNA-LNPs. Data are presented as mean ± SEM; *p<0.05. (**D**) *In vivo* bioluminescence imaging at 24 h post-injection of mRNA-LNPs in C57BL/6 mice. mRNA-LNPs was injected intramuscularly in the TA at the dose of 1 µg. Color scale indicates radiance (photons/s/cm²/sr). (**E**) Luciferase activity in TA

and liver at 24 h shown as RLU/µg of protein. n=3; One-way ANOVA followed by Fisher's LSD test; Data are presented as mean ± SEM; *p<0.05 **p<0.01.

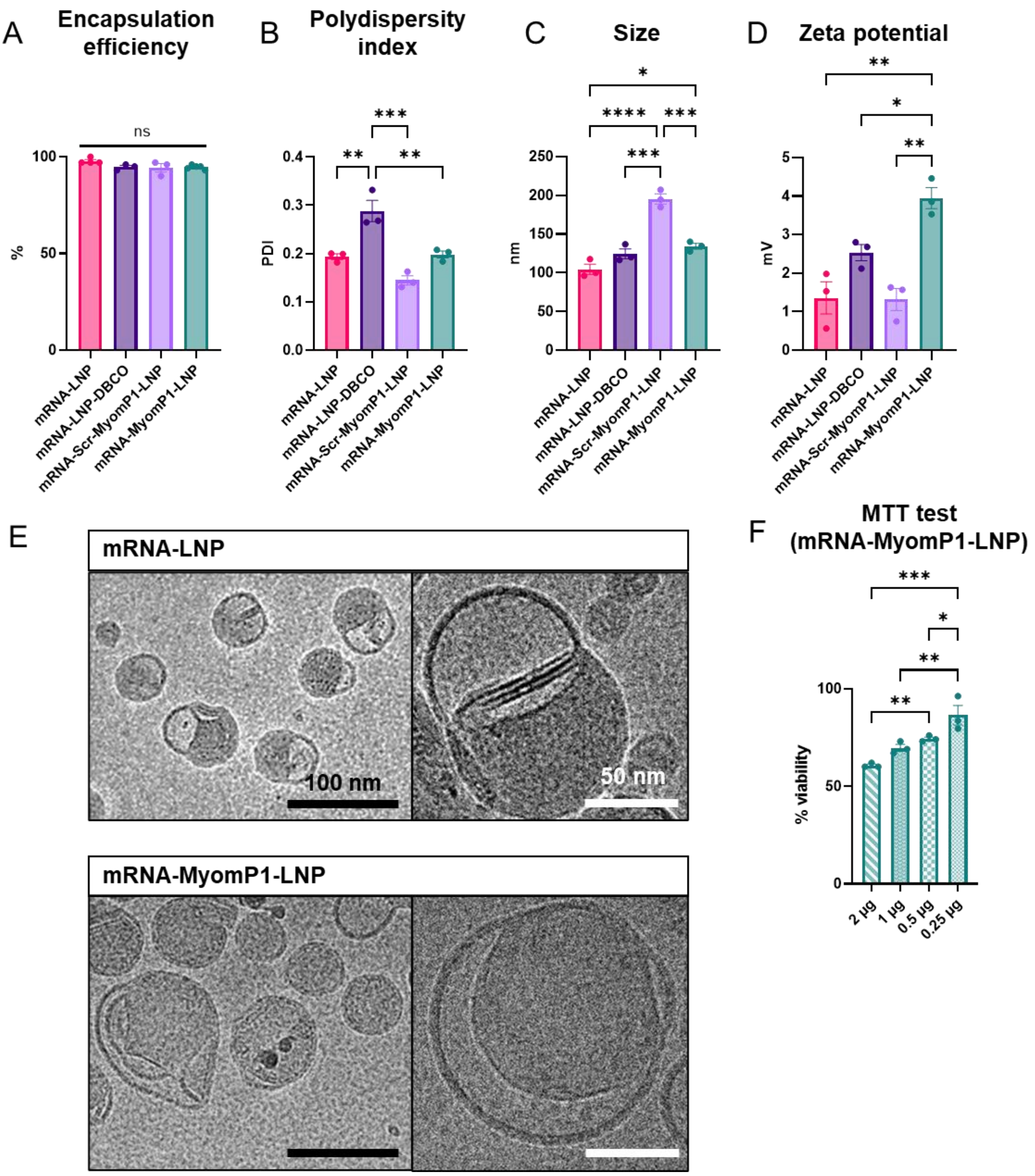

Figure S3. **Characterization of peptide-functionalized mRNA-LNPs**. **(A)** Encapsulation efficiency of mRNA-LNPs. **(B)** Polydispersity index of mRNA-LNPs. **(C)** Size of mRNA-LNPs. **(D)** Zeta potential of mRNA-LNPs. The zeta potential was measured at pH=7 in PBS buffer. **(E)** Morphology of mRNA-LNPs. Cryo-electron microscopy images were generated to

visualize mRNA-LNP morphology. Black scale bars represent 100 nm and 50 nm. (**F**) Cell viability in myoblasts after transduction with different doses of mRNA-MyomP1-LNP. n=3; One-way ANOVA followed by Fisher's LSD test; Data are presented as mean ± SEM; *p<0.05 **p<0.01, ***p<0.001, ****p<0.0001.

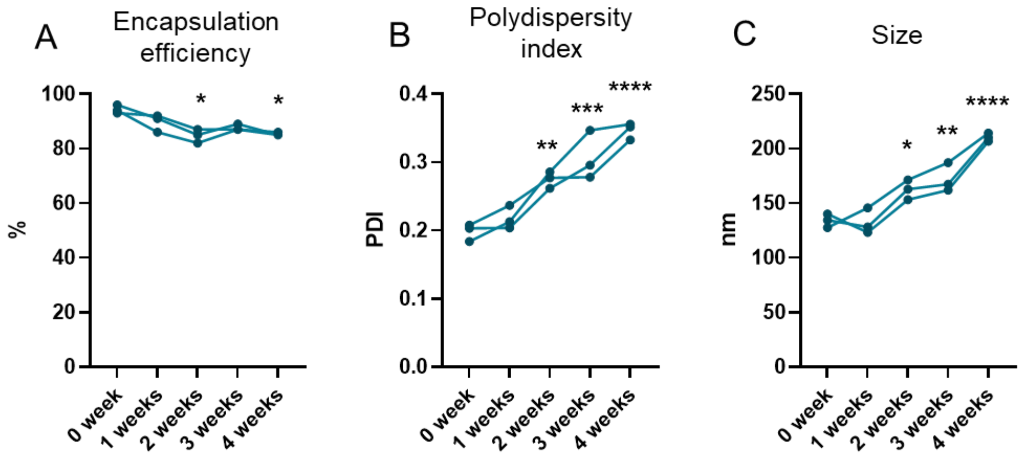

Figure S4. Evaluation of the stability of mRNA-MyomP1-LNP over weeks at 4°C. The encapsulation efficiency, polydispersity index, and particle size of mRNA-MyomP1-LNPs were evaluated weekly for up to four weeks during storage at 4°C. **(A)** Encapsulation efficiency of mRNA-MyomP1-LNPs. **(B)** Polydispersity index of mRNA-MyomP1-LNPs. **(C)** Size of mRNA-MyomP1-LNPs. n=3; One-way ANOVA followed by Fisher's LSD test; Data are presented as mean ± SEM; *p<0.05 **p<0.01, ***p<0.001, ****p<0.0001; *vs 0 week.

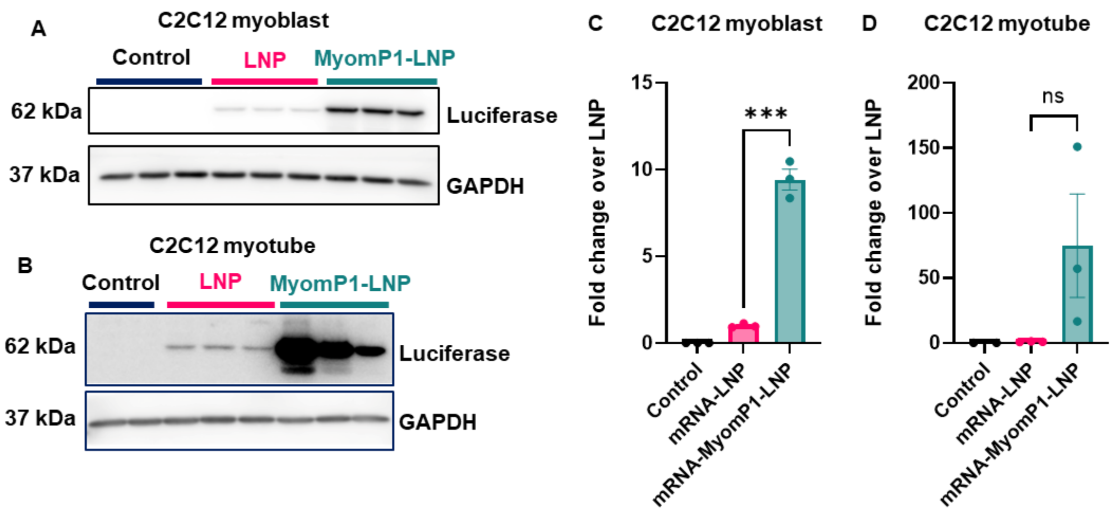

Figure S5. Luciferase protein level in C2C12 myoblasts and myotubes transfected with mRNA-LNPs. (A,B) Luciferase protein expression in transfected-C2C12 myoblasts (**A**) and myotubes (**B**). **(C,D)** Protein level was normalized with GAPDH expression. n=3; One-way ANOVA followed by Fisher's LSD test or Krustal-Wallis test followed by Dunn's LSD test; Data are presented as mean ± SEM; ***p<0.001.

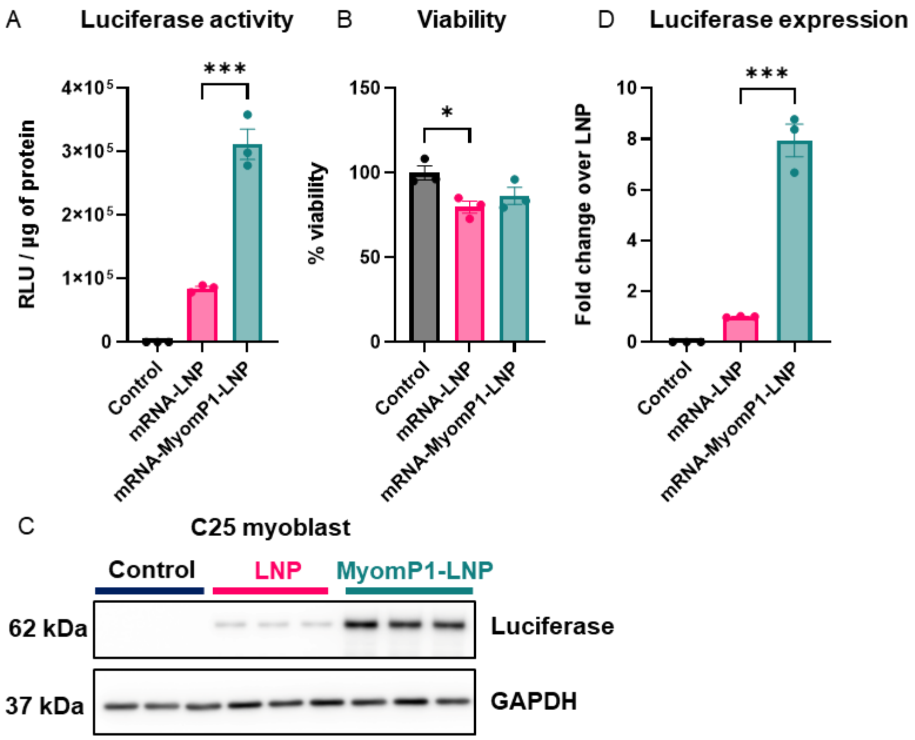

Figure S6. ***In vitro* transfection efficacy and cell viability of peptide-functionalized mRNA-LNPs in human myoblasts. (A)** Luciferase activity in C25 human myoblasts shown as relative luminescence units (RLU) per µg of total protein. **(B)** Viability of C25 human myoblasts post-transduction. **(C,D)** Luciferase protein expression in transfected C25 human myoblasts. Protein level was normalized with GAPDH expression. n=3; Data are presented as mean ± SEM; One-way ANOVA followed by Fisher's LSD test; *$p < 0.05$, ***$p < 0.001$.

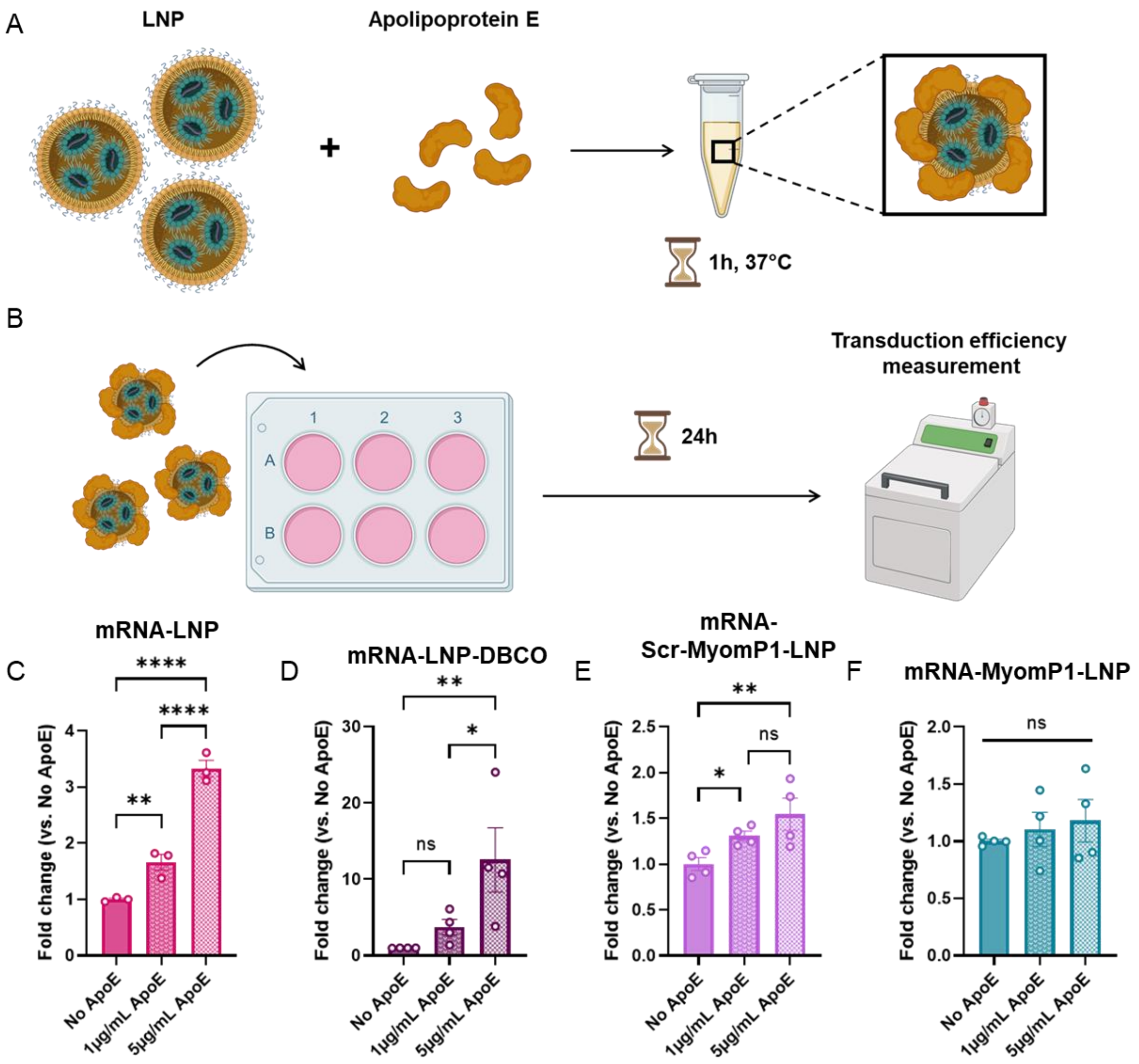

Figure S7. Impact of ApoE on mRNA-LNP transduction efficiency. (**A**) Schematic representation of ApoE corona formation on the surface of LNPs. (**B**) Illustration of the *in vitro*

protocol in C2C12 myoblasts to assess the effect of ApoE on transduction efficiency. (C-F) Luciferase activity in C2C12 myoblasts after incubation with mRNA-LNPs in the absence or presence of ApoE at different doses (1 or 5 µg/mL) for: (C) mRNA-LNP, (D) mRNA-LNP-DBCO, (E) mRNA-Scr-MyomP1-LNP, and (F) mRNA-MyomP1-LNP. Luciferase activity is expressed as fold change relative to the No ApoE condition. Data represent mean ± SEM, n = 3-4; One-way ANOVA followed by Fisher's LSD test; *$p < 0.05$, **$p < 0.01$, ****$p < 0.0001$.

Table S1. Lipid composition of LNPs

Lipid	Stock solution (mg/mL)	Molar ratio	CAS number	Reference
Dlin-KC2-DMA	100	50	1190197-97-7	MedChemExpress, #HY-112758
SM-102	100		2089251-47-6	MedChemExpress, #HY-134541
1,2-DSPC	25	10	816-94-4	MedChemExpress, #HY-W040193
Cholesterol	5	38.5	57-88-5	MedChemExpress, #HY-N0322
DMG-PEG2000	1	1.4	160743-62-4	MedChemExpress, #HY-112764
DSPE-PEG2000-DBCO	1	0.1	2052955-83-4	Avanti, #880229

Table S2. Primers used for genes encoding immune-related markers and the housekeeping gene

Gene	Forward	Reverse
Il-6	ACAACCACGGCCTTCCCTACTT	CACGATTTCCCAGAGAACATGTG
Irf7	CCCATCTTCGACTTCAGCAC	TGTAGTGTGGTGACCCTTGC
Ccl2	AGGTCCCTGTCATGCTTCTG	AAGGCATCACAGTCCGAGTC
Cxcl10	CCAAGTGCTGCCGTCATTTTC	TCCCTATGGCCCTCATTCTCA
Rps11	CGCGTGGTGAATAAGGAAGC	GTAAGCACGCTCCGTCTGAA

Table S3. Antibodies used in flow cytometry

Name	Format	Clone	Supplier	Reference	Dilution
CD4	PE	RM4-5	BD Biosciences	553049	1/100e
CD8	Alexa 700	53-6.7	BD Biosciences	557959	1/100e
CD44 (Pgp-1)	FITC	IM7.8.1	BD Biosciences	561859	1/100e
CD62L	PE-CF594	MEL-14	BD Biosciences	562404	1/100e
CD19	RB705	1D3	BD Biosciences	570651	1/200e
B220	Pe-Cy7	RAR3-6B2	e-Bioscience	25-0452	1/100e
